# Sequential recruitment of body fluid spaces for increasing volumes of crystalloid fluid

**DOI:** 10.3389/fphys.2024.1439035

**Published:** 2024-08-28

**Authors:** Robert G. Hahn

**Affiliations:** Department of Clinical Sciences at Danderyd Hospital, Karolinska Institutet, Stockholm, Sweden

**Keywords:** crystalloid fluid, pharmacokinetics, hemodilution, statistics and numerical data, water-electrolyte balance, physiology, blood loss, general anesthesia

## Abstract

**Introduction:**

The interstitial space harbours two fluid compartments linked serially to the plasma. This study explores conditions that lead to fluid accumulation in the most secluded compartment, termed the “third space”.

**Methods:**

Retrospective data was collected from 326 experiments in which intravenous crystalloid fluid was administered to conscious volunteers as well as a small group of anaesthetized patients. The urinary excretion and plasma dilution derived from haemoglobin served as input variables in nine population volume kinetic analyses representing subtly different settings.

**Results:**

An infusion of 250–500 mL of Ringer’s solution expanded only the central fluid space (plasma), whereas the infusion of 500–1,000 mL extended into a rapidly exchanging interstitial fluid space. When more than 1 L was infused over 30 min, it was distributed across plasma and both interstitial fluid compartments. The remote space, characterized by slow turnover, abruptly accommodated fluid upon accumulation of 700–800 mL in the rapidly exchanging space, equivalent to an 11%–13% volume increase. However, larger expansion was necessary to trigger this event in a perioperative setting. The plasma half-life of crystalloid fluid was 25 times longer when 2,000–2,700 mL expanded all three fluid compartments compared to when only 250–500 mL expanded the central space (14 h versus 30 min).

**Conclusion:**

As the volume of crystalloid fluid increases, it apparently occupies a larger proportion of the interstitial space. When more than 1 L is administered at a high rate, there is expansion of a remote “third space”, which considerably extends the intravascular half-life.

## Background

Crystalloid electrolyte solutions serve as the cornerstone of fluid therapy in surgery and trauma treatments. Their kinetics are complex, making it challenging to predict their effect on intravascular volume. In volunteers the infusion expands plasma volume by roughly 20% following distribution (Moore et al., 1966; Jacob et al., 2012) but the expansion increases to 50% for the duration of the infusion (Hahn, 2020a). General anaesthesia significantly delays excretion due to decreased arterial pressure (Hahn, 2017). Both propofol and volatile anaesthetics suppress lymphatic pumping (Bachmann et al., 2019) and vasoactive signal molecules may increase the retention of fluid in the interstitial space (Dull and Hahn, 2023; Dargent et al., 2023).

The term “third-spacing”, which refers to the abnormal distribution of fluids, is somewhat ambiguous. While the existence of a “third fluid space” remains disputed (Shires et al., 1961; Jacob et al., 2009), crystalloid fluids can apparently be sequestered into an inaccessible interstitial space (Hahn, 2020b). This phenomenon may account for the observed increase in body weight up to a week post-major surgery, even in the absence of indications of vascular overload (Brandstrup et al., 2003; Cihoric et al., 2023).

In the present study, filling of the “third fluid space” is demonstrated to be a regular event once a certain amount of crystalloid has entered the fluid space immediately adjacent to the bloodstream. Importantly, this event will be shown to prolong the half-life of the infused fluid in the body by several multiples. The analysis is based on recent kinetic findings proposing that filtered fluid collects in two compartments: a fast-exchange interstitial fluid compartment (*V*_t1_) and a slower-exchange compartment (*V*_t2_), of which the latter might be referred to as the “third fluid space” (Hahn and Dull, 2024). These may possibly be analogous to the free fluid phase and the gel phase within the interstitium (Haljamäe, 1978; Aukland and Reed, 1993). However, it has been unclear if there is a parallel or sequential (serial) exchange with the plasma. According to a new analysis, the exchange appears to be sequential (Hahn et al., 2023).

The aim of the present study was to investigate the conditions that allow the “third fluid space” to fill during crystalloid therapy when the plasma is serially connected with the interstitial compartments. The endpoint was fluid accumulation in the two interstitial fluid spaces based on population kinetic analysis. These accumulations were compared in nine groups of volunteers and patients who received different volumes of Ringer´s solution or were in a special condition (general anaesthesia, blood loss, or acute inflammation). The hypothesis was that the remote “third space” becomes filled in some groups but not in others, and that prerequisites are possible to identify.

## Methods

The study material came from a database of intravenous infusion experiments in humans that aimed to examine fluid volume kinetics. The author planned and analysed these experiments, employing similar standardized protocols and sample collection methods. They were performed between 1993 and 2017 at Karolinska University Hospital (Huddinge, Sweden), Södersjukhuset (Stockholm, Sweden), Linköping University Hospital (Linköping, Sweden) and Shaoxing People´s Hospital (Shaoxing, China).

### Inclusion criteria and ethical approvals

Data was taken from 326 experiments drawn from 13 studies, all of which included routine measurements of blood haemoglobin concentrations during and after the intravenous infusion of crystalloid fluid administered a constant rate via infusion pumps. A comprehensive list of the studies included, as well as the details of the ethics approvals and the experiments, can be found in Supplementary Material. Complete case series were used, i.e., no originally studied patient was excluded, but only the control experiments were used when a study included deliberate dehydration or used colloid fluid. Surgeries performed in a Trendelenburg position were excluded because changes in body position from the flat recumbent may alter the crystalloid fluid kinetics (Hahn, 2017; Hahn and Dull, 2024). The exclusion criteria further ruled out individuals < 18 years and those with severe cardiac, lung, hepatic, or renal disease.

All study protocols received approval from the relevant Ethics Committees, and the research was conducted per the Declaration of Helsinki. Prior to participating in any experiment, every subject provided their written, informed consent. Reporting followed the STROBE statement checklist.

### Procedures

Before they arrived at the hospital, volunteers were permitted to consume a sandwich and a glass of liquid to avert dehydration and hunger stress. Patients planned for general anaesthesia were required to fast, though they were administered 5 mg of diazepam orally as a premedication. The experiments commenced between 8 and 9 a.m. with an initial 30-min bedrest to attain a hemodynamic steady state. Subjects remained in the flat recumbent position throughout the experiments. Two intravenous cannulas were positioned in each arm’s cubital vein, one for blood sampling and the other for consistently regulated infusion via a pump. Blood was drawn every 5 min during the infusion and for an additional 30 min after that, then again at intervals of 15–30 min for a total duration ranging from 1.5 to 6 h, though typically around 3–4 h. The blood’s haemoglobin concentration was assessed at the onsite clinical chemistry laboratory, with a variation coefficient of roughly 1%. While lying supine, the volunteers urinated in a bucket as needed. Urine produced during surgery was collected through a continuous bladder catheter and measured to the nearest 25 mL.

The infused crystalloid fluids were Ringer’s acetate (N = 278), Ringer’s lactate (N = 34), and 0.9% saline (N = 10). The Ringer’s solutions have identical volume kinetics (Hahn, 2017), while the slower elimination of 0.9% saline was adjusted to match Ringer’s using a covariate analysis of *k*_10_ (Hahn et al., 2016). No fluid other than the study solution was provided. Monitoring procedures included pulse oximetry, electrocardiogram, and non-invasive arterial pressure.

### Study groups

Six subgroups of normohydrated healthy volunteers received increasing volumes of crystalloid fluid, usually over 30 min. The 7^th^ group consisted of patients undergoing open hysterectomy or thyroid resection under general anaesthesia with minimal bleeding. The 8^th^ group was 10 healthy volunteers who underwent two infusion experiments, one being initiated just after withdrawal of 450 mL of blood and the other after withdrawal of 900 mL of blood (mean, 650 mL). The 9^th^ subgroup included of patients who were operated due to acute appendicitis or cholecystitis; contributed data were derived from both the awake and anaesthetized states. These subgroups are further detailed in Table 1.

**TABLE 1:** Basic data for the studied subgroups and the experiments.

Fluid group	Infu-sions (N)	Age (years)	Ma-les (%)	Body Weight (kg)	Hb (g/L)	Infused volume (mL)	Infusion time (min)	Rate (mL/min)	Data points (N)	Urine collections (N)	Akaike crite-rion
250–500 mL	37	44 (21)	78	79 (12)	131 (13)	397 (242)	15	26 (4)	388	37	*
500–1,000 mL	24	28 (7)	70	74 (12)	132 (9)	856 (127)	31 (14)	33 (15)	507	56	*
1,000–2,000	67	30 (7)	75	73 (9)	133 (13)	1,762 (248)	30	59 (8)	1952	81	−11278
2,000–2,700	38	30 (7)	100	88 (6)	139 (8)	2,170 (143)	30	72 (5)	1,153	81	−4,254
“Fast” infusions	29	32 (6)	66	79 (14)	139 (13)	1,941 (342)	15	125 (22)	516	41	−1,532
“Extended” infusions	17	33 (7)	47	70 (12)	131 (9)	1,321 (355)	51 (14)	26 (7)	384	17	*
Anaesthesia	54	50 (12)	8	71 (12)	120 (3)	1,779 (316)	30	59 (11)	930	210	−2,438
Haemorrhage	20	28 (12)	100	75 (7)	—	1,841 (136)	30	61 (5)	463	20	−1,599
Inflammation	41	44 (12)	40	58 (11)	—	869 (168)	35	25 (5)	753	40	−1,122

Continuous data are reported as the mean (standard deviation).

* Number of compartments was dependent on whether statistically significant parameter estimates could be obtained.

### Kinetic analysis

The method of study was population (mixed models) kinetics, which a standard industry procedure for analysing drug turnover. However, we employed fluid-induced haemodilution as input data (dependent variable) rather than the traditional method of relying on plasma drug concentration (Hahn, 2020a). The haemodilution concept was used because the plasma concentration of an infusion fluid cannot be measured, while its volume can be indicated by observing the dilution of molecules already present in the blood. Haemoglobin is particularly useful as it only remains in the bloodstream and is easy to measure accurately.

**FIGURE 1:**
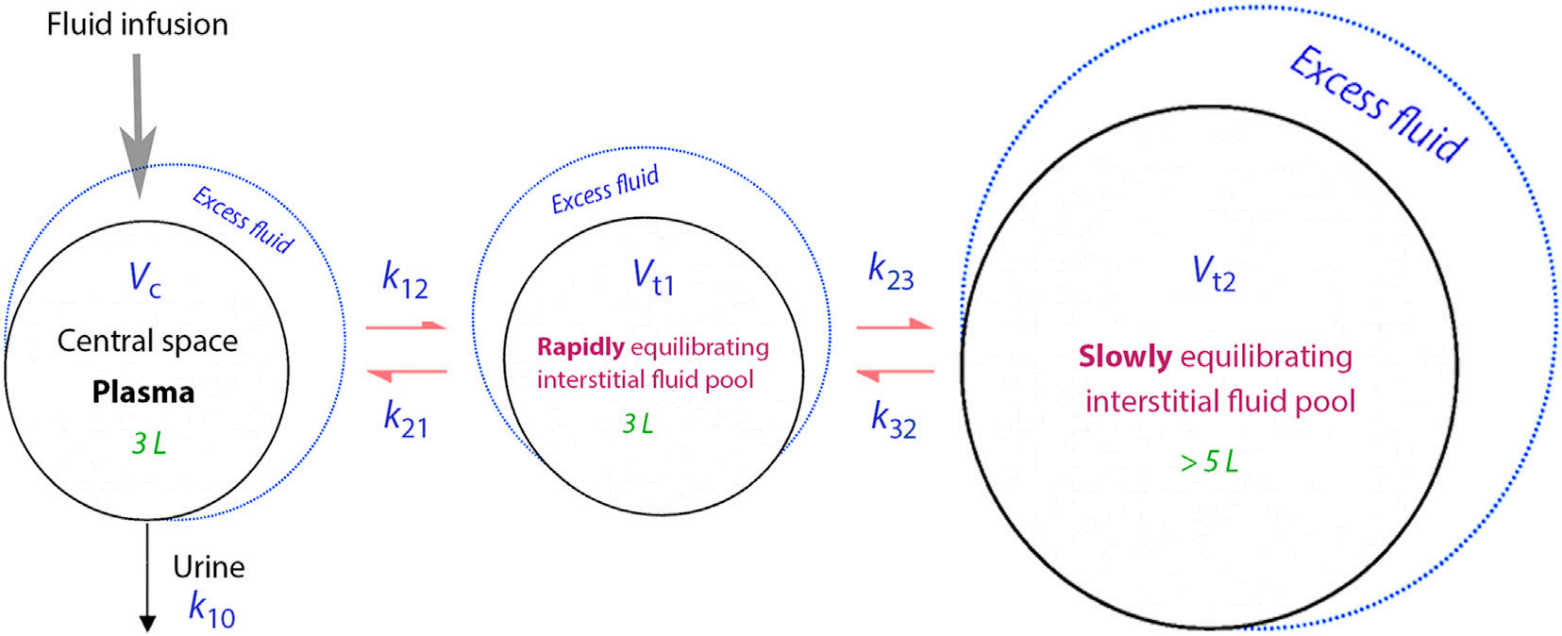
Schematic drawing of the kinetic model used for the study.

Figure 1 presents a schematic drawing of the kinetic model, while the differential equations can be found in Supplementary Material. The model employs five fixed-rate constants (*k*_12_, *k*_21_, *k*_23_, *k*_32_ and *k*_10_) to dictate the fluid flow between three body fluid spaces, also known as “compartments” (*V*_c_, *V*_t1_ and *V*_t2_). *V*_c_ signifies the operational plasma volume. *V*_t1_, the fast-exchange interstitial compartment, which sits between *V*_c_ and *V*_t2_, the slow-exchange compartment, which only communicates with *V*_t1_. As a result, infused fluid needs to be filtered to *V*_t1_ before it can reach *V*_t2_. The elimination rate constant, *k*_10_, is taken as the measured urine output divided by the area under the curve of the excess volume in *V*_c_.

The five parameters in the kinetic model were fitted to two dependent variables by a computer program using log-likelihood mathematics, Phoenix software (version 8.3.4) designed for nonlinear mixed effects (Phoenix NLME, Pharsight, St. Louis, MO). The first dependent variable was the fractional plasma dilution, calculated as haemodilution divided by (1 – baseline haematocrit), which then signified the expansion of *V*_c_ from the infusion. The second dependent variable was the measured urine output.

The flow between compartments were the product of the fixed-rate constant signifying the flow (Figure 1) and the volume expansion of the body fluid compartment from where the flow originated. Hence, the flow varied over time in proportion to the filling of that fluid space. However, the model´s five fixed-rate constants (*k*_12_, *k*_21_, *k*_23_, *k*_32_, and *k*_10_) and *V*_c_ could be modified by individual-specific *covariates*, such as body weight and sex. In this study, we concentrated on the presumed relationship between body weight and *V*_c_ and on time-dependent inhibition of rate constants controlling flow rates (Hahn, 2024). Urine volumes and a detailed explanation of how covariates are mathematically incorporated into the kinetic model can be found in Supplementary Material.

The kinetic analysis was conducted in 9 subgroups because it may not be justifiable to include all three fluid compartments in every study setting. Whether a 2-volume or 3-volume model was statistically justified was evaluated by the Akaike criterion. The choice could also be limited by lack of convergence or statistically significant output parameters. Data from each subgroup was analysed using the Phoenix software, using the First Order Conditional Estimation Extended Least Squares technique (FOCE ELS) as search routine.

The optimal estimates of the model parameters in each subgroup were inserted into the simulation module of the Phoenix program to predict the volume expansion of each fluid compartment (being one, two, or three) every minute up to 180 min in response to the mean infused volume. Longer follow-up times (days) could be used for *post hoc* exploratory analyses.

The plasma half-life of the fluid was determined by simulating the volume distribution extracted from the Phoenix program and calculating the time needed for a 50% reduction in plasma volume expansion (*V*_c_). The whole-body half-life was obtained in a similar way but after summarizing the excess fluid in all compartments (*V*_c_ + *V*_t1_ +*V*_t2_).

The behaviour of the model (goodness of fit) was studied by comparing the measured with the predicted plasma dilution values, illustrated by predictive checks and plots of the predicted plasma dilution *versus* the conditional weighted residuals (CWRES). These plots are shown for all subgroups in the Supplementary Material.

## Results

The basic data for the 9 cohorts is detailed in Table 1. Plasma dilution was recorded on 7,046 instances across 326 experiments, with an average of 22 measurements per experiment.

**FIGURE 2:**
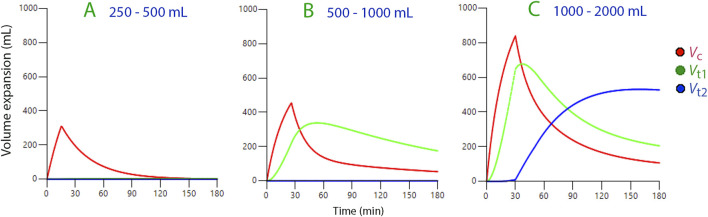
Volume expansion of body fluid compartments based on kinetic data derived from three ranges of infused volumes of Ringer’s solution. Symbols: 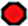 *V*_c_ 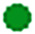 *V*_t1_ 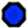 *V*_t2_ **(A)** 250–500 mL over 15 min where only the central space (*V*_c_) is filled. **(B)** 500–1,000 mL over 30 min where fluid is distributed to the fast-exchange interstitial space, *V*_t1_. **(C)** 1,000–2,000 mL over 30 min, where fluid is also distributed to the slow-exchange interstitial space, *V*_t2_. The infusion volumes used for the simulations were the mean of the infusion volumes used to derive the kinetic parameters (397, 855, and 1,762 mL, respectively). Plots of the volume expansion over time in all subgroups are shown in the Supplementary Material.

When we infused Ringer’s solution, ranging from 250 to 500 mL, into conscious volunteers, it only expanded the central fluid compartment (the plasma), which we estimated to average 3.46 L in size (N = 37; Figure 2A).

Infusions between 500 and 1,000 mL were distributed between the central compartment and the fast-exchange peripheral space, *V*_t1_ (N = 24; Figure 2B).

We noted that after a delay, volumes greater than 1,000 mL also extended to a slow-exchange peripheral space, *V*_t2_ (the “third fluid space”, N = 105; Figure 2C).

**FIGURE 3:**
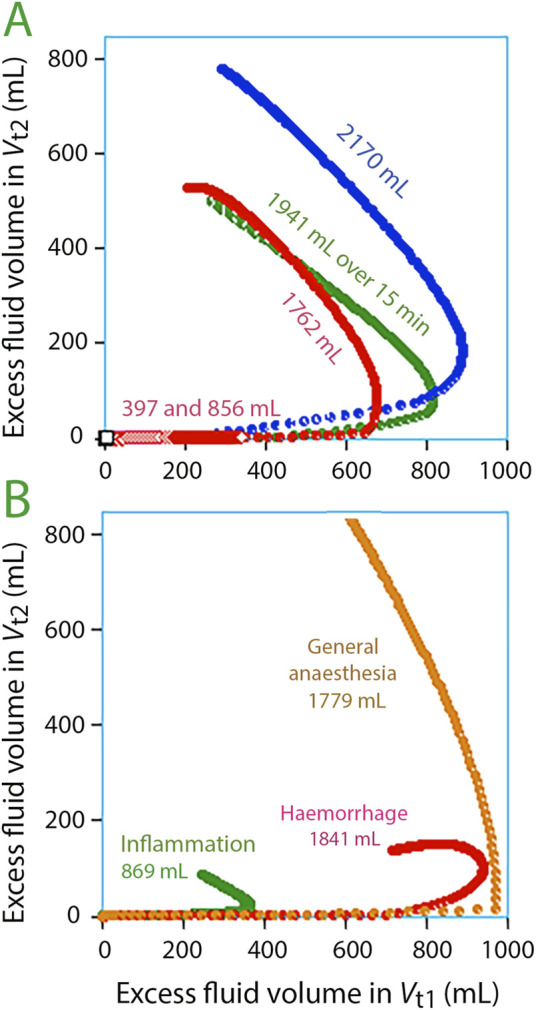
Volume expansion of *V*_t1_ *versus V*_t2_ every minute during 3 h. The edge of the curve indicates that fluid begins to accumulate in *V*_t2_. The optimal model parameters for each subgroup were used for the simulations. **(A)** Experiments in awake volunteers. **(B)** Experiments in special settings.

Rapid replenishment of *V*_t2_ occurred once the volumetric expansion of *V*_t1_ reached 700–800 mL (Figure 3A). These events corresponded with infusion durations of 15 and 30 min. However, there was no noticeable filling of *V*_t2_ when an average volume of 1,321 mL was dispensed over a period ranging from 45 to 80 min, averaging 51 min (“extended” infusions; N = 17), but *V*_t1_ increased only by 600 mL *V*_t2_ during these experiments.

Figure 3B illustrates fluid infusions under specific conditions. Filling of *V*_t2_ was greatest during general anaesthesia but was occurred at a slightly larger expansion of *V*_t1_ compared to volunteers. After haemorrhage, Ringer’s caused only minor filling of *V*_t2_ (N = 20). For patients with inflammation, *V*_t1_ appeared to require less expansion before *V*_t2_ filling began (N = 40).

**FIGURE 4:**
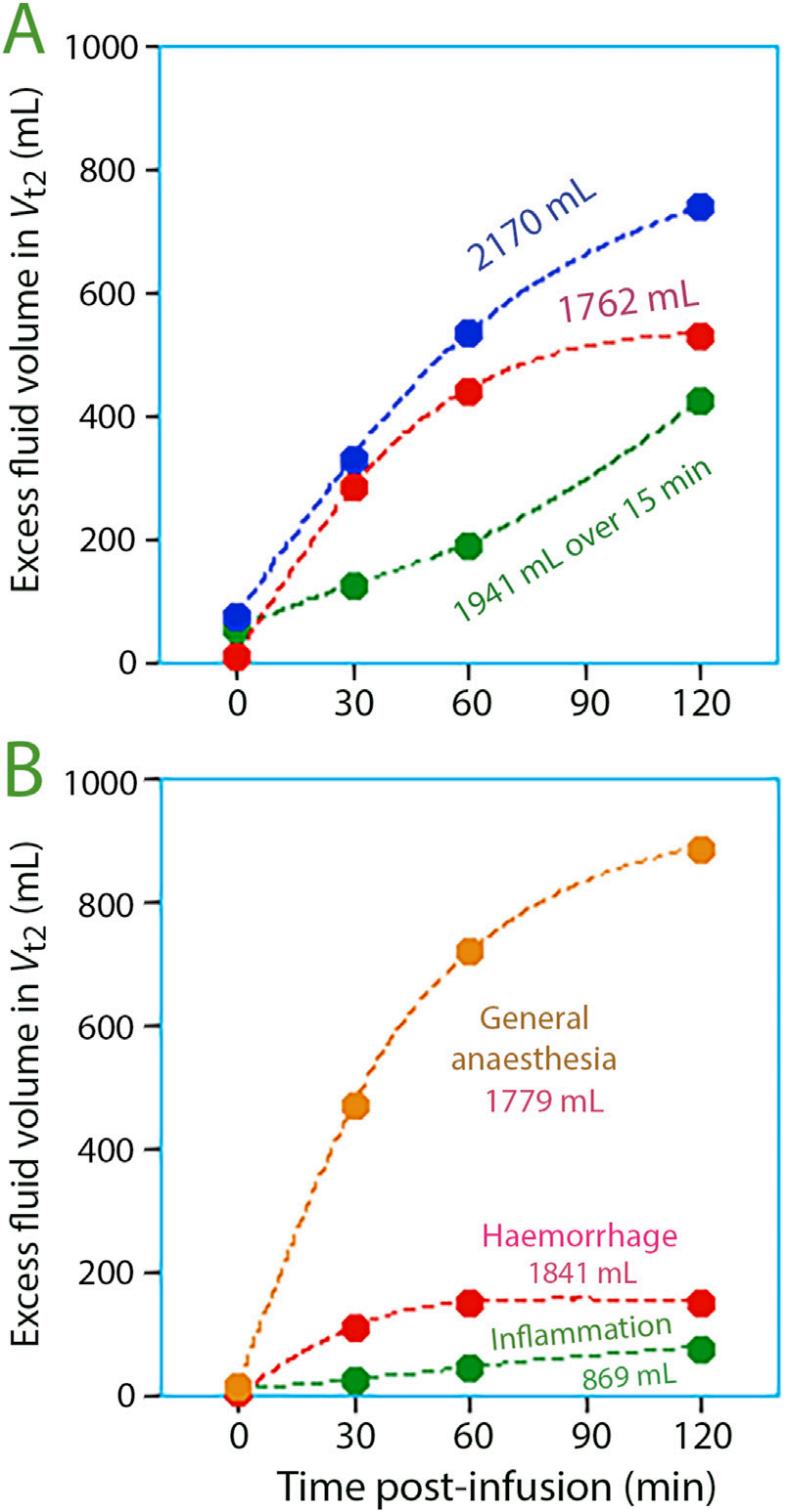
Volume expansion of the slow-exchange interstitial compartment, *V*_t2_, at various points in time post-infusion for **(A)** three study groups with volunteers, and **(B)** three study groups studied in special settings.

The fluid build-up in *V*_t2_ was negligible at the end of infusion but progressively became significant over time (Figure 4).

**FIGURE 5:**
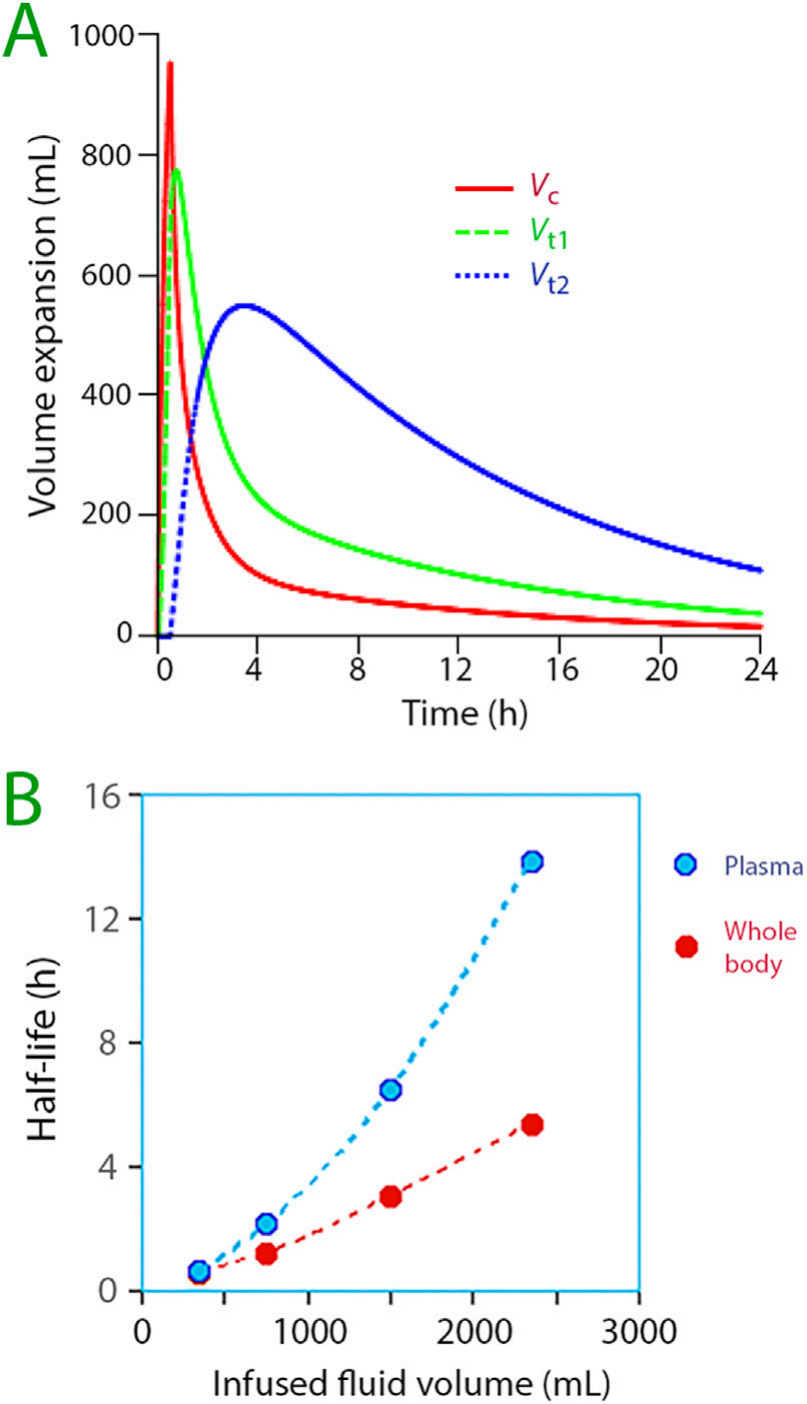
**(A)** Distribution of infused fluid between the three body fluid compartments when 1,800 mL of Ringer´s is infused in awake volunteers. The optimal parameter estimates for 67 infusions of 1,000–2,000 mL of fluid was used for the simulation. Symbols: 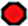 *V*_c_ 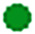 *V*_t1_ 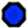 *V*_t2._ **(B)** The half-life for Ringer solution in the plasma (*V*_c_) and the whole body (*V*_c_ + *V*_t1_ +*V*_t2_) for increasing infused volumes. The simulated numerical volumes were based on the optimal parameter estimates for each fluid volume range and is plotted *versus* the middle value of each range. Symbols: 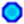
Plasma 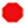
Whole body.

Simulations extending the observation time indicated that the “third space” was the largest fluid compartment amplified upon infusion of more than 1 L of Ringer’s solution (Figure 5A). This considerably prolonged both the infused fluid’s half-life in the body as well as the half-life of the plasma volume expansion (Figure 5B).

The kinetic parameters for all cohorts are detailed in Supplementary Material, which also includes a simulation plot for each cohort.

The mean arterial pressure mainly differed between the awake and anesthetized subjects; the means (standard deviations) were 88 (9) and 77 (15) mmHg, respectively, for 1,294 and 1,509 measurements (unpaired *t*-test, *P*< 0.001).

## Discussion

### Key findings

When increasing amounts of crystalloid fluid are infused, fluid compartments appear to be subsequently added in a specific sequence. A minor elevation in capillary pressure is apparently necessary to distribute fluid to the fast-exchange interstitial fluid space (*V*_t1_). When more than 1 L of Ringer’s solution is administered at a speed exceeding 30 mL min^-1^, fluid build-up occurs in an isolated slow-exchange interstitial fluid area (*V*_t2,_ “third fluid space”). This compartment’s filling starts sharply, subsequently becoming the main repository for the infused fluid. This substantial fluid accumulation markedly extends the half-life of the fluid. Excess volume will linger in the body for several days if the kinetic parameters remain unchanged over time.

### What opens the “third space”?

This report is the first to assess prerequisites for entrance of crystalloid fluid to the remote “third space” when the two interstitial compartments are connected in the physiologically correct way (Hahn et al., 2023). A comprehensive analysis was set aside in preference for subgroup analyses since it is possible that the “third space” may not be discernible in all circumstances. The differences between the study groups have not been possible to disclose based on the underlying studies alone.

The results show that *V*_t1_ needs to be filled 700–800 mL to generate enough interstitial pressure for fluid to move into *V*_t2_ in volunteers, which represents a volume increase of *V*_t1_ by 11%–13% (Figure 3A). This concept of a threshold is further confirmed by longer infusion times; lower levels of *V*_t1_ expansion did not trigger the opening of *V*_t2_.

Guyton and colleagues showed, in 1966, that the compliance for interstitial volume expansion is very low when the pressure is subatmospheric [normally 3–7 mmHg below the atmospheric pressure, depending on the tissue (Guyton et al., 1971)]; however, the compliance increases more than 100,000-fold when the pressure is equal to or higher than the surrounding air, indicating a suddenly increased size of tissue spaces (Guyton et al., 1966; Guyton and Coleman, 1968). Hence, it is possible that *V*_t2_ opened when the fluid infusions increased the interstitial pressure enough to make it exceed the surrounding air pressure. The threshold appeared to be slightly different in special conditions, which might be due to deviating interstitial pressures at baseline (Hopkinson et al., 1968) (Figure 3B).

No suction pressure holds the tissues together when the interstitial pressure becomes higher the surrounding air, which means that tissues may break up. Animal experiments with isotonic saline and irrigating fluids support that severe overhydration damages the cytoarchitecture and promotes the formation of lacunae (pools of fluid) in connective tissue and organs, including the heart (Hahn et al., 1996; Hahn et al., 2000). Although a speculation at present, it is possible that the “third-spacing” developing already after infusing 2–3 L of crystalloid fluid in humans causes morphological changes that disturbs organ function. This is a concern that needs to be studied, as it has not yet been made.

A regular observation in the present study was a significant inhibitory effect on the filling of *V*_t2_ during the ongoing infusion. Various mathematical analyses were conducted to challenge this inhibition with the goal of questioning whether the end of the infusion was indeed the true initiation point for *V*_t2_ filling. Smaller volumes of fluid could potentially enter *V*_t2_ at earlier intervals, as exemplified in the largest subgroups. However, the primary fluid entry into *V*_t2_ still occurred at the end of the infusion (Figure 3). Consequently, it is plausible that the suction effect that is expected to develop when fluid is no longer added to an already accelerated flow across *V*_t1_ may have played an additive role when *V*_t2_ opened for fluid accumulation.

### Why is there a “third space”?

The concept of the “third” fluid space, introduced in the 1960s (Shires et al., 1961), suggests that larger than usual volumes of fluid should be administered during surgery and intensive care (Choudhuri et al., 2023). Some scholars dispute its existence (Jacob et al., 2009). Nonetheless, it is observed that liberal fluid administration easily leads to increased body weight for several days post-major surgery (Brandstrup et al., 2003; Cihoric et al., 2023).

The current kinetic analysis enhances our understanding of the associated events. Fluid build-up in *V*_t2_ can account for the prolonged oedema that forms after quickly infusing crystalloid fluid. This response occurs both in volunteers and during surgery. The fluid displacement to *V*_t2_ moderates fluid overload more effectively than distribution to *V*_t1_ alone because the fluid is returned to the plasma at a very low rate.

The volume expansion potential for *V*_t2_ appears to be great. Estimations of compartment sizes derived from the kinetic parameters and suggest that *V*_t1_ typically occupies a baseline volume of 6–7 L. Conversely, *V*_t2_ often exhibits supraphysiological dimensions, implying restricted rather than free-flowing movement. This suggests that fluid embedded herein faces difficulties in being expelled. Lack of free exchange is illustrated in Figure 5B by the increasing half-life when progressively more fluid is infused in and, above all, by the marked difference between the plasma half-life and the whole-body half-life.

*V*_t1_’s physiological correlates may comprise the combined water channels of the interstitium’s free fluid phase and the lymphatic vessels, whereas *V*_t2_ likely aligns with the gel phase (Haljamäe, 1978; Aukland and Reed, 1993). The latter compartment is chiefly made up of proteoglycan filaments that limit water mobility. The free fluid phase functions similarly to a telephone line, given that increased capillary fluid leakage swiftly escalates the lymphatic flow within the thoracic duct in mere minutes (Brace and Power, 1983). Conversely, in the gel phase, water volume transport happens via molecular diffusion rather than chain reactions (Guyton and Hall, 1996).

### Kinetic consequences

“Third-spacing” had limited effect on the fluid distribution during a short 30-min infusion and the subsequent 30-min distribution phase (Figure 5A). Its role as safety factor that prevents intravascular overload did not become important until after the period of the greatest plasma volume expansion, being initiated when 700–800 mL of fluid had filled *V*_t1_. This means that a long infusion time can limit “third-spacing” as more of the infused fluid has then had time to become excreted; this assumption was also corroborated by the “extended infusions” in the present study. However, this preventive effect requires a prompt diuretic response to plasma volume expansion, which may not be at hand. The diuretic response to crystalloid fluid loading is inhibited by low arterial pressure (Hahn, 2017; Taavo et al., 2021), old age (Hahn, 2017), elevated plasma creatinine (Hahn, 2021), blood loss and dehydration (Hahn et al., 2020), and even by high urine osmolality due to low habitual intake of water (Hahn et al., 2020). Several of these effects are mediated via hormones, such as vasopressin and aldosterone (Perrier et al., 2013; Armstrong et al., 2020; Norberg et al., 2007), and the adrenergic nervous system (Taavo et al., 2021). Given the strong inhibition of the diuretic response to crystalloid fluid due to low arterial pressure during general anaesthesia [−90% (Hahn, 2017)] a longer infusion time is unlikely to prevent “third-spacing” in that setting.

In the present study, the diuretic response to volume loading was quantified by the kinetic parameter *k*_10_, which is reported as tvKe for all nine subgroups in the Supplementary Material. This parameter averaged 0.023 min^−1^ for the infusions performed in awake volunteers while being only 0.002 min^−1^ during general anaesthesia (9% of the value in the awake state).

The most apparent kinetic consequence of “third-spacing” for the relatively short infusion times in the present study was a marked prolongation of the half-life of infused fluid. This effect is illustrated in Figure 5B but becomes even more clear when comparing long and short infusions of the same crystalloid volume in awake volunteers. For example, the whole-body half-life was 31 min with the “extended infusion” (average 51 min). In contrast, an exploratory analysis found a half-life of 290 min for infusions of nearly the same volume (average 1,358 mL) given over 30 min, which effectively opened *V*_t2_ (N = 14, data not shown). Only in this second group was the three-volume model statistically justified (Akaike criterion). The difference represents close to a 10-fold prolongation of the half-life.

### Clinical implications

The full scenario of the clinical implications of “third-spacing” cannot be given at present because the possibility to characterize this compartmentation is a recent finding. “Third-spacing” is likely to be a main factor explaining the long-lasting increase in body weight (up to 1 week) observed after major surgery (Brandstrup et al., 2003; Cihoric et al., 2023). Furthermore, liberal administration of crystalloid fluid during surgery increases the incidence of postoperative adverse events, most of which arise from the lungs (oedema and infection), gastrointestinal tract (break up of sutures, sepsis), and the skin (poor wound healing) (Arieff, 1999; Nisanevich et al., 2005; Joosten et al., 2018). However, the contribution of “third-spacing” to the development of these complications is unclear.

Blood loss is known to lower the interstitial pressure (Hopkinson et al., 1968) and the autotransfusion of interstitial fluid to the plasma that follows the induction of general anaesthesia performed without fluid loading (Dull and Hahn, 2021) can both be expected to require greater filling of *V*_t1_ before “third-spacing” occurs; indeed, this is also what was found in the present study.

The smaller volume expansion of *V*_t1_ required for “third-spacing” to be initiated in the patients with acute inflammation may be relevant for several medical diseases. This finding indicates a pre-existing overfilling of the extravascular space, which might have occurred due to a suction effect created by interstitial accumulation of cytokines and other inflammatory mediators (Wiig et al., 2003; Dull and Hahn, 2023).

### Limitations

The mathematical calculations applied to plasma dilution accounted for blood loss, including surgical bleeding and sampling (Löffel et al., 2021; Ewaldsson and Hahn, 2005). However, they did not consider the impact of evaporation. Insensible fluid losses total 30 mL h^-1^, but only 10 mL per h^−1^ would be drawn from the kinetic system being studied due to its derivation from total body water. In addition, the initial values for capillary leakage rate and lymphatic return were not reported. It is, however, reasonable to assume these steady-state flows can reach a rate of 7 mL min^-1^ (Hahn and Dull, 2024).

The data utilized was extracted from a repository of infusion experiments designed to investigate volume kinetics. The varying sampling protocols only influenced the duration of these experiments, which typically lasted from 3 to 4 h but could extend up to 6 h in some instances. Most of this data has been employed in previous studies. The data from volunteer subjects were gathered from a specific age range, bodyweight category, and haemoglobin concentration level. The surgical patients were somewhat older and were randomly assigned to receive either intravenous or volatile anaesthetics (Ewaldsson and Hahn, 2005; Hahn and Nemme, 2020).

A few subgroups contain repeat infusions; the volunteers who participated in the haemorrhage experiments underwent two infusions and those receiving 1.0–2.7 L could be given Ringer´s acetate on one occasion and Ringer’s lactate on another. Patients experiencing inflammation were scrutinized pre-, post-, and during anaesthesia. Laparoscopic surgeries were omitted due to their kinetics that differs from operations performed in recumbent position (Hahn, 2017; Hahn and Dull, 2024).

This study comprises groups with mean ages spanning from 28 to 50 years, weighing 58–88 kg, and including 8%–100% of males (Table 1). Previous work shows that old age (Hahn, 2017) and male sex (Hahn, 2016) is associated with slightly poorer diuretic response to volume loading which should, in theory, facilitate development of “third-spacing”. However, a concrete effect cannot yet be proposed as “third-spacing” seems to be greatly dependent on the interstitial pressure at baseline as well.

## Conclusion

The interstitial space comprises two fluid compartments. Infusion experiments with crystalloid fluid in volunteers, analysed using population volume kinetics, reveal that fluid rapidly permeates into a remote slow-exchange interstitial fluid space (*V*_t2_). This occurs when capillary filtration has expanded a fast-exchange interstitial fluid space (*V*_t1_) by about 11%–13%, typically equating to 700–800 mL of fluid. A larger expansion of *V*_t1_ may be necessary in a perioperative setting to open up *V*_t2_, but filling was more extensive than in the volunteers. Over time, this remote space becomes the largest expanded compartment, significantly extending the half-life of the infused fluid.

## Data Availability

The original contributions presented in the study are included in the article/Supplementary Material. Further inquiries can be directed to the corresponding author.
