# Supplementary material for: Sequential recruitment of body fluid spaces for increasing volumes of crystalloid fluid
Source: Front Physiol. 2024 Aug 28;15:1439035. doi: 10.3389/fphys.2024.1439035 (PMC11387178; doi:10.3389/fphys.2024.1439035)
Supplement: Supplementary file 1 [file DataSheet1.docx]

­­­­­­­­­

**Supplementary File**

Sequential recruitment of body fluid spaces for increasing volumes of crystalloid fluid

**_________________________________________________________________________**

**Content**

1. Table S1, list of manuscripts that provided data for the analyses
2. Detailed list of the included studies
3. Kinetic model
4. Covariate analysis
5. 250-500 mL; volume plot and kinetic output
6. 500-1,000 mL; volume plot and kinetic output
7. 1,000-2000 mL; volume plot and kinetic output
8. Graphical performance measures for the first 3 subgroups
9. 2,000-2,700 mL; volume plot and kinetic output
10. 15-min infusions of > 1 L; volume plot and kinetic output
11. Infusion duration 45-80 min; volume plot and kinetic output
12. Graphical performance measures for the second series of 3 subgroups
13. General anesthesia; volume plot and kinetic output
14. Hemorrhage; volume plot and kinetic output
15. Inflammation; volume plot and kinetic output
16. Graphical performance measures for the last series of 3 subgroups
17. Tabulated performance measures
18. Phoenix program file

________________________________**________________________________________**

**Table S1.** The manuscripts that provided data for the analysis, with ethics approvals.

| Males /  females | Infusions | Surgery | Publication | Ethics approval | Committee |
| --- | --- | --- | --- | --- | --- |
|  |  |  |  |  |  |
| 10 / 0 | 30 | No | Anesthesiology 1999, 90, 81-91. | 54/95 | Huddinge Hospital |
| 10 / 0 | 36 | No | Br J Anaesth 1999, 82, 496-502 | 54/95 | Huddinge Hospital |
| 8 / 0 | 8 | No | Br J Anaesth 2001: 87: 834-43 | 276/96 | Huddinge Hospital |
| 10 / 0 | 30 | No | Anesthesiology 2002, 96, 1371-80 | 228/98 | Huddinge Hospital |
| 10 / 0 | 17 | No | Anesth Analg 2012; 115; 814-22 | M114-09 | Linköping |
| 0 / 9 | 9 | No | Acta Anaesthesiol Scand 2011, 55, 987-94 | 123/97 | Huddinge Hospital |
| 10 / 0 | 10 | No | Crit Care 2013, 17, R104 | 2009/1091-31/2 | Stockholm |
| 19 / 8 | 27 | Just before surgery | Acta Anaesthesiol Scand 2014, 58, 1258-66 | 2011/101-31 | Linköping |
| 4 / 25 | 29 ^1^ | Thyroid surgery | Anesthesiology 2005, 103, 460-9 | 269/02 | Huddinge Hospital |
| 0 /25 | 25 | Open hysterectomy | BMC Anesthesiology 2020, 20: 95 | 2016-01-27 ^2^ | Riga Stradins, Latvia |
| 0 / 6 | 30 | No | Br J Anaesth 1997, 78, 144-8 | 168/91 | Huddinge Hospital |
| 21 / 16 | 37 | Appendictomy, cholecystectomy | Br J Anaesth 2018, 121, 574-80 | 2015013 | Shaoxing Hospital, P. R. China |
| 19 / 1 | 34 | No | No separate publication | 115–00 + 168/91  2007/851-31/4 | Huddinge Hospital +  Stockholm |
|  |  |  |  |  |  |

The country is Sweden except where noted.

^1^ one patient with major hemorrhage was excluded.

^2^ the date of the approval is given as no numbering was practiced.

**2. Detailed list of the studies providing data for the analyses**

1. Drobin D, Hahn RG. Volume kinetics of Ringer’s solution in hypovolemic volunteers. Anesthesiology 1999:90:81–91.
2. Hahn RG, Drobin D, Ståhle L. Volume kinetics of Ringer’s solution in female volunteers. Br J Anaesth 1997: 78: 144–148.
3. Drobin D, Hahn RG. Kinetics of isotonic and hypertonic plasma volume expanders. Anesthesiology. 2002:96:1371–1380.
4. Hahn RG, Nemme J. Volume kinetic analysis of fluid retention after induction of general anaesthesia. BMC Anesthesiology 2020; 20: 95.
5. Sjöstrand F, Edsberg L, Hahn RG. Volume kinetics of glucose solutions given by intravenous infusion. Br J Anaesth 2001: 87: 834–843.
6. Svensén C, Drobin D, Olsson, J, Hahn RG. Stability of the interstitial matrix after crystalloid fluid loading studied by volume kinetic analysis. Br J Anaesth 1999: 82: 496–502.
7. Zdolsek J, Li Y, Hahn RG. Detection of dehydration by using volume kinetics. Anesth Analg 2012; 115; 814–822.
8. Hahn RG, Lindahl C, Drobin D. Volume kinetics of acetated Ringer’s solution during experimental spinal anesthesia. Acta Anaesthesiol Scand 2011; 55: 987–994.
9. Hahn RG, Bergek C, Gebäck T, Zdolsek J. Interactions between the volume effects of hydroxyethyl starch 130/0.4 and Ringer’s acetate. Crit Care 2013; 17: R104.
10. Hahn RG, Bahlmann H, Nilsson L. Dehydration and fluid volume kinetics before major open abdominal surgery. Acta Anaesthesiol Scand 2014; 58: 1258–1266.
11. Ewaldsson CA, Hahn RG. Kinetics and extravascular retention of acetated Ringer’s solution during isoflurane and propofol anesthesia for thyroid surgery. Anesthesiology 2005:103:460–469.
12. Hahn RG, Nemme J. Volume kinetic analysis of fluid retention after induction of general anaesthesia. BMC Anesthesiology 2020; 20: 95.
13. Li Y, Yi S, Zhu Y, Hahn RG. Volume kinetics of Ringer’s lactate solution in acute inflammatory disease. Br J Anaesth 2018; 121: 574–580.

**3. Kinetic model.**


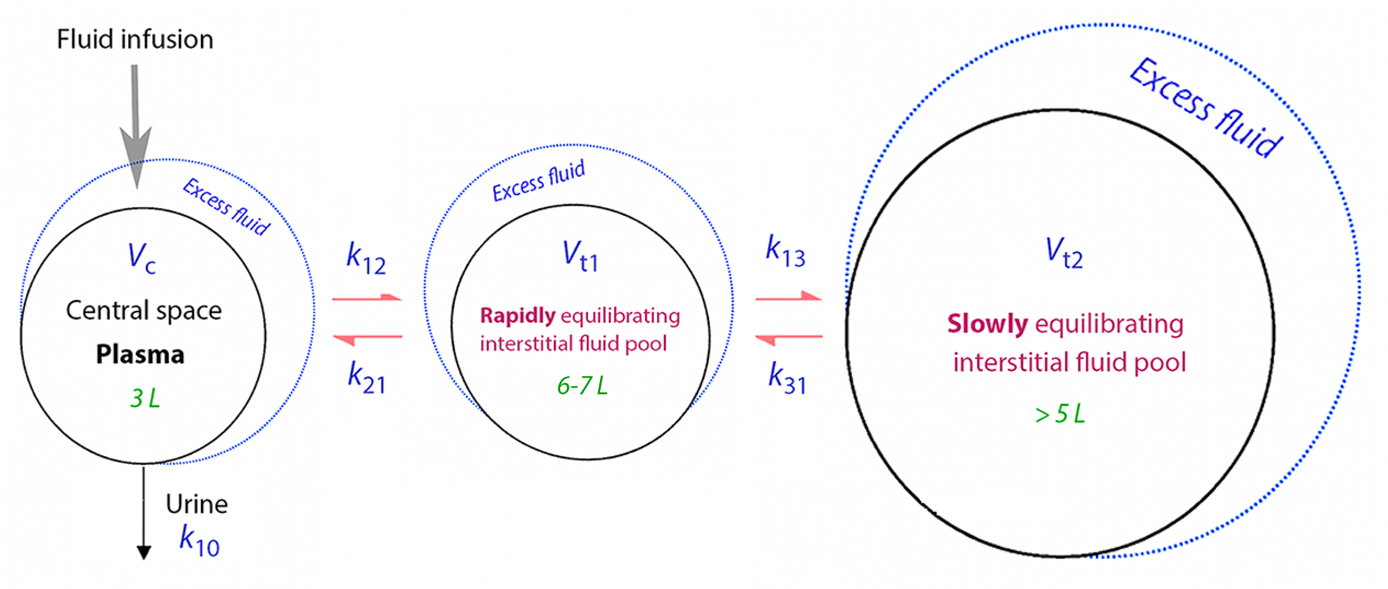


The description below relates to **Fig. S1** above. Fluid is infused at the rate *R*_o_ into the plasma, *V*_c_, from which distribution occurs at a rate determined by a rate constant *k*_12_ to a rapid-exchange interstitial space, *V*_t1_. The distributed fluid returns to *V*_c_ at a rate determined by a constant *k*_21_. Fluid is also distributed from *V*_t1_ to a slow-exchange interstitial space, *V*_t2_, at a rate determined by a constant *k*_23_. The return of this fluid to *V*_t1_ is governed by a rate constant *k*_32_. Urinary excretion (U) occurs in proportion to the expansion of *V*_c_ by a rate constant *k*_10_. Hence, the two interstitial compartments are connected to *V*_c_ in a serial fashion. The differential equations are:

 d*v*_c_ /dt = *R*_o_ – *k*_12_ (*v*_c_ – *V*_c_) + *k*_21_ (*v*_t1_ – *V*_t1_) – *k*_10_ (*v*_c_ – *V*_c_)

d*v*_t1_ /dt = *k*_12_ (*v*_c_ – *V*_c_) – *k*_21_ (*v*_t1_ – *V*_t1_) – *k*_23_ (*v*_t1_ – *V*_t1_) + *k*_32_ (*v*_t2_ – *V*_t2_)

d*v*_t2_ /dt = *k*_23_ (*v*_t1_ – *V*_t1_) – *k*_32_ (*v*_t2_ – *V*_t2_)

dU /dt = *k*_10_ (*v*_c_ – *V*_c_)

Expanded volumes are indicated by lower-case letters (*v*_c_, *v*_t1,_ and *v*_t2_) and baseline volumes and variables by capital letters (*V*_c_, *V*_t1,_ and *V*_t2_).

The hemodilution [(Hb/hb)–1] was divided by (1– baseline hematocrit) to obtain the Hb-derived fractional plasma dilution, which corresponds to (*v*_c_ – *V*_c_) / *V*_c_. The excreted urine, whenever collected, is used as input variable for *U*. The glycocalyx volume is part of *V*_c_ [Intensive Care Med Exp 2020; 8: 29].

**4. Covariate models**

The rate constants and *V*_c_ could all be modified by *covariates*, which are characteristics that may change the parameter estimates in a specific individual. The most promising candidates for covariate effects was searched by plots of random effects ("eta:s"). The promising variables were then added one by one to the model and accepted if the -2 log likelihood (-2 LL) for the model then decreased by > 6.6 points (*P*< 0.01).

In the present study, a commonly found covariate was a positive relationship between the *V*_c_ and the body weight, which is expected as the infused fluid volume was given in proportion to the body weight. This correction was made by using a *power model* which is appropriate for positive continuous data. For example, if the covariate effect is reported is 1.6, the body weight in a specific individual is 80 kg but the mean for all subjects is 73 kg and the optimal estimate of *V*_c_ for all subjects is 3,000 mL, the size of *V*_c_ in the specific individual is given by:

*V*_c_ = 3,000 * [(80 / 73) ^1.6^ ] = 3,473 mL

Other covariates were inhibition of *k*_21_ during the infusion and a reduction of the entrance of fluid to *k*_23_ for predefined time periods during and after the infusion. These covariate effects were evaluated by the *exponential covariate model* which is appropriate for categorical variables. For example, the rate parameter *k*_23_ for volunteers receiving 1,000 mL-2,000 mL of Ringer´s over 30 min had the group value of 0.017 min^-1^ but covariate effect of -3.5 applied to the period between 20 and 30 min of the infusion, the value equation for *k*_23_ becomes:

*k*_23_ = 0.017 [e ^--3.5^] = 0.00051

as e = 2.718. The value 0.00051 relates to the period between 20 and 30 min of the infusion while 0.017 is valid for other time periods of the experiment, except if a significant covariate effect is found for another time period.

**5. 250-500 mL (awake)**

37 infusions; age 44 (21), Sex 78% males, BW 79 (12)

Hb 131 (13) inf vol 397 (242). Inf time 15 min, rate 26 (4) mL min^-1^

388 data points. 37 urine outputs, total urine volume 332 (244) mL.

Data are the mean (SD).


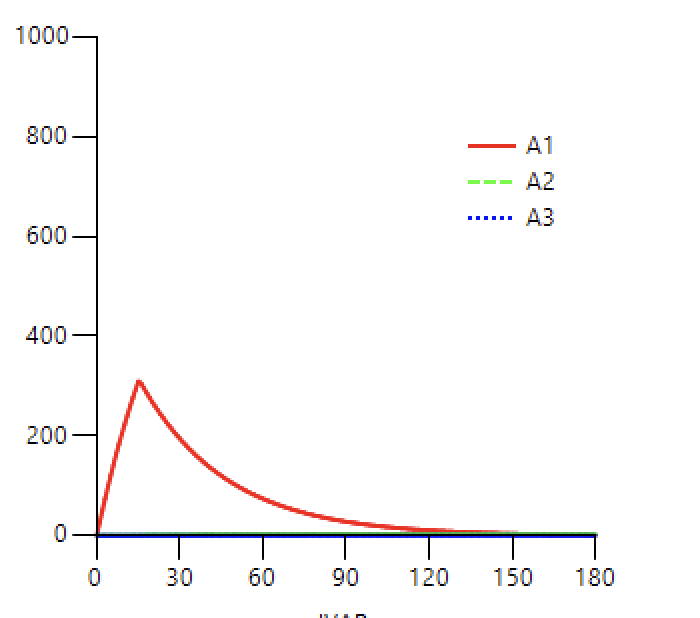


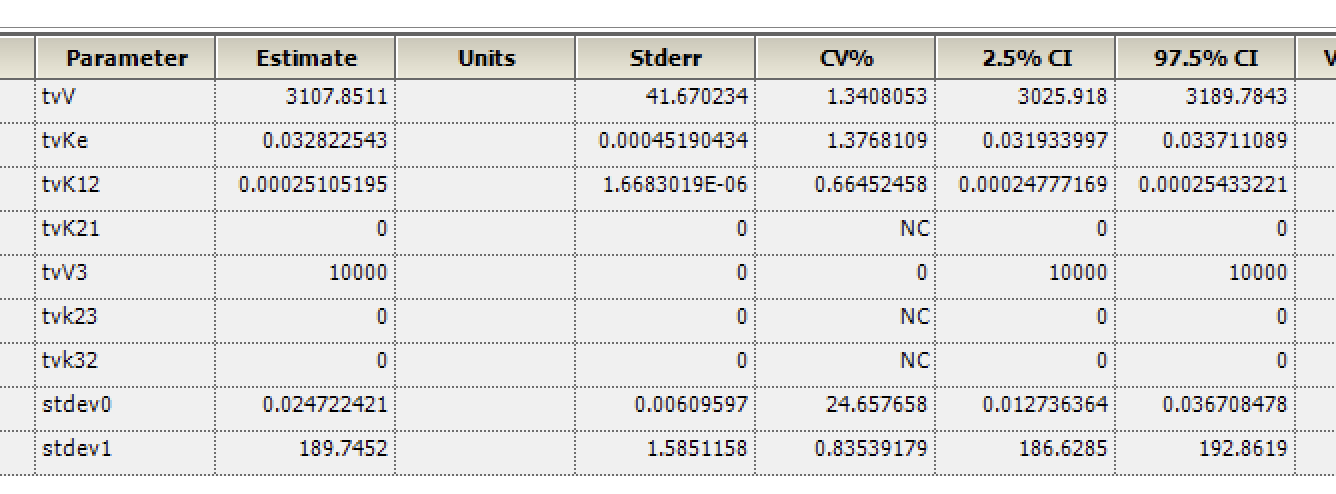


Kinetic parameters above are given as the best estimate and 95% confidence interval.

**6. 500-1000 mL (awake)**

23 infusions; age 28 (7), Sex 70% males, BW. 74 (12)

Hb 132 (9) inf vol 856 (127). Inf time 31 (14) min, rate 33 (15) mL min^-1^

507 data points. 56 urine outputs, total urine volume 548 (237) mL.

Data are the mean (SD).

DV = volume expansion, IVAR = time (min), Red color = *V*_c_, Green color = *V*_t1_,


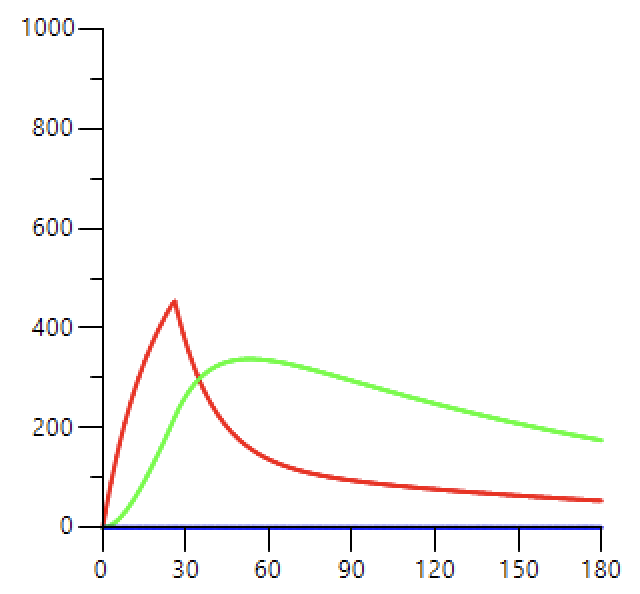

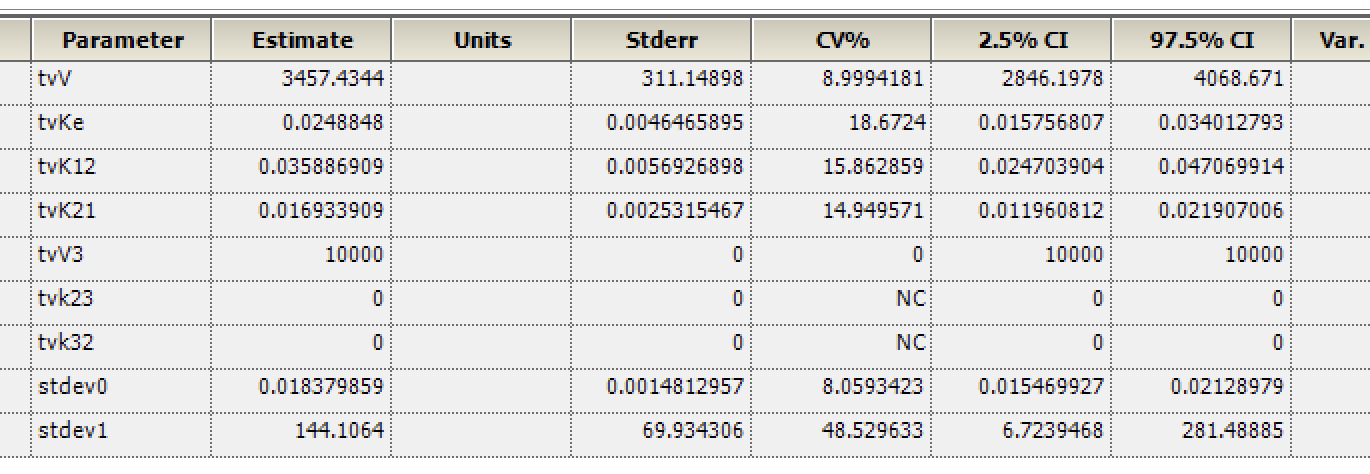


**7. 1000-2000 mL (awake)**

*Only 30-min infusions included*

67 infusions; age 30 (7), Sex 75% males, BW 73 (9) kg.

Hb 133 (13) inf vol 1762 (248). Inf time 30 min, rate 59 (8) mL min^-1^

Data are the mean (SD).

1952 data points. 81 urine outputs, total urine volume 922 (370) mL.

Akaike criterion 3-volume model -11278 and 2-volume model -7206


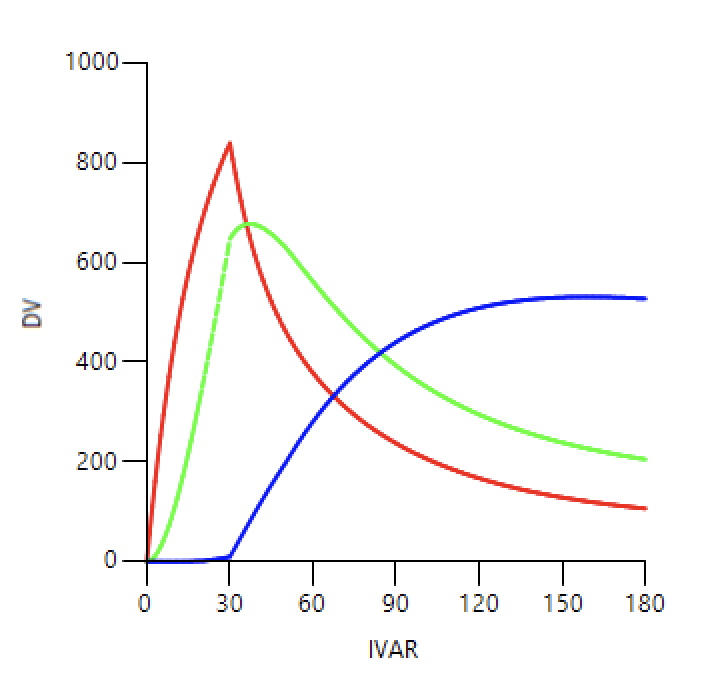


DV = volume expansion, x-axis = time (min), Red color = *V*_c_, Green color = *V*_t1_, Blue color = *V*_t2_


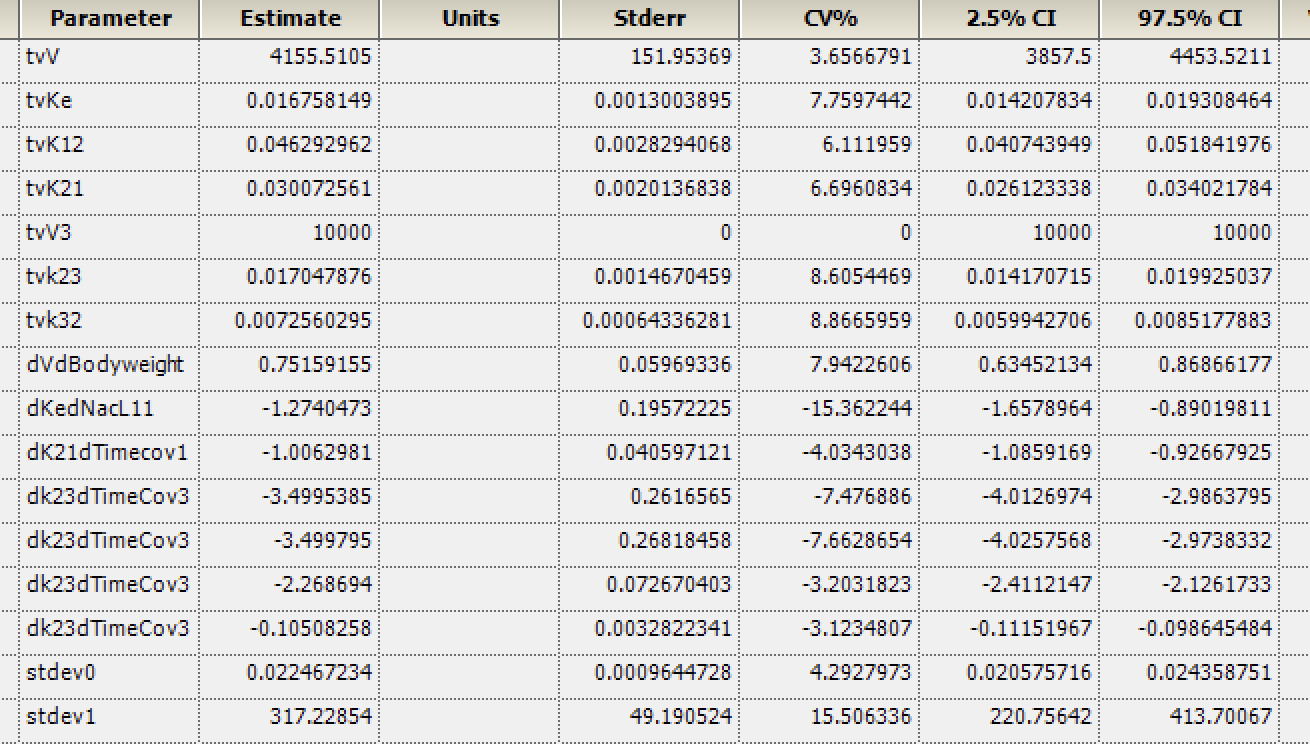


Time Cov 1: during infusion time. Time cov 3 relates to (in order from top): first 15 min of infusion, 15-20 min of infusion, 25-30 min. of infusion, 0-15 min after the end of the infusion.

**
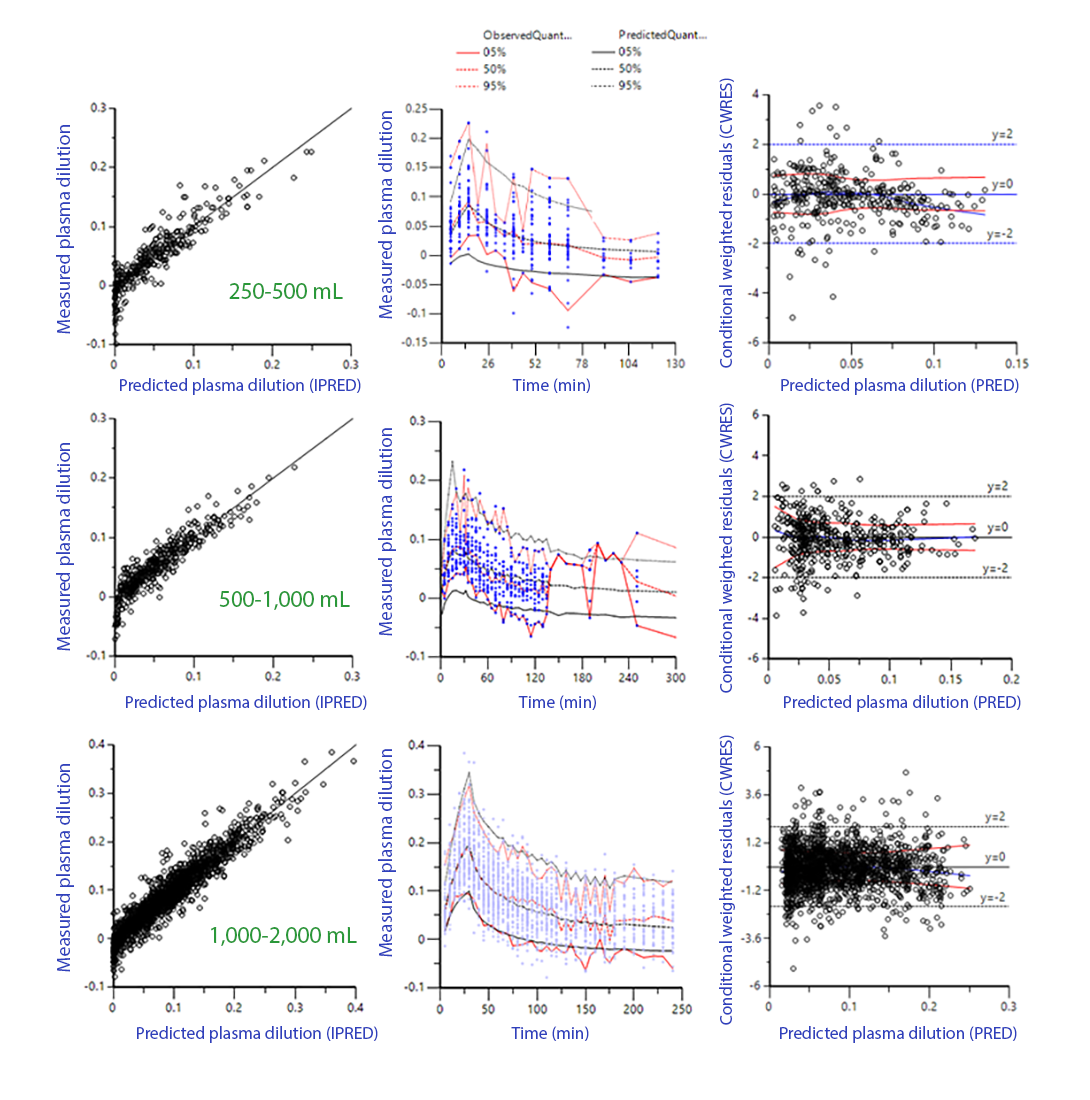
**

**8. GRAPHICAL PERFORMANCE MEASURES 1**

top: 250-500 mL, middle: 500-1,000 mL, and bottom:1,000-2,000 mL

**Left column:** Measured plasma dilution *versus* the predicted dilution (with covariates).

**Middle column:** Predictive check. The measured plasma dilution and its 95% confidence interval is compared to the confidence interval for 1,000 simulations based in the optimal model parameters.

**Right column:** The conditional weighted residuals (CWRES) *versus* the predicted plasma dilution (without covariates).

**9. 2000-2700 mL (awake)**

*Only 30 min infusions.*

38 infusions; age 30 (7), Sex 100% males, BW. 88 (6)

Hb 139 (8) inf vol 2170 (143). Inf time 30 min, rate 72 (5) mL min^-1^

Data are the mean (SD).

1153 data points. 81 urine outputs, total urine volume 1013 (428) mL.

Akaike criterion 3-volume model -4254 and 2-volume model -4227


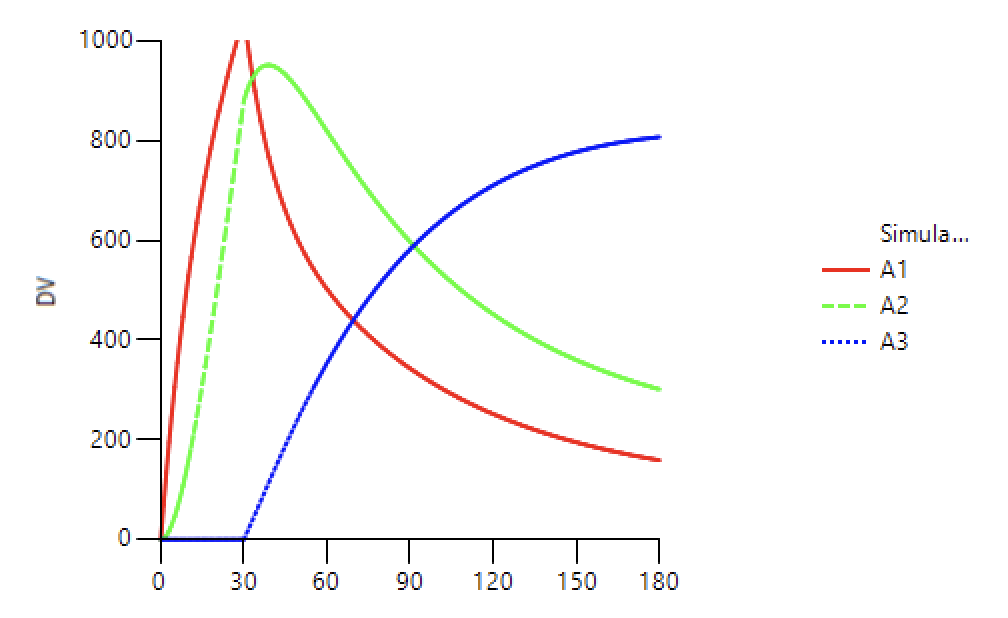


DV = volume expansion, x-axis = time (min), Red color = *V*_c_, Green color = *V*_t1_, Blue color = *V*_t2_


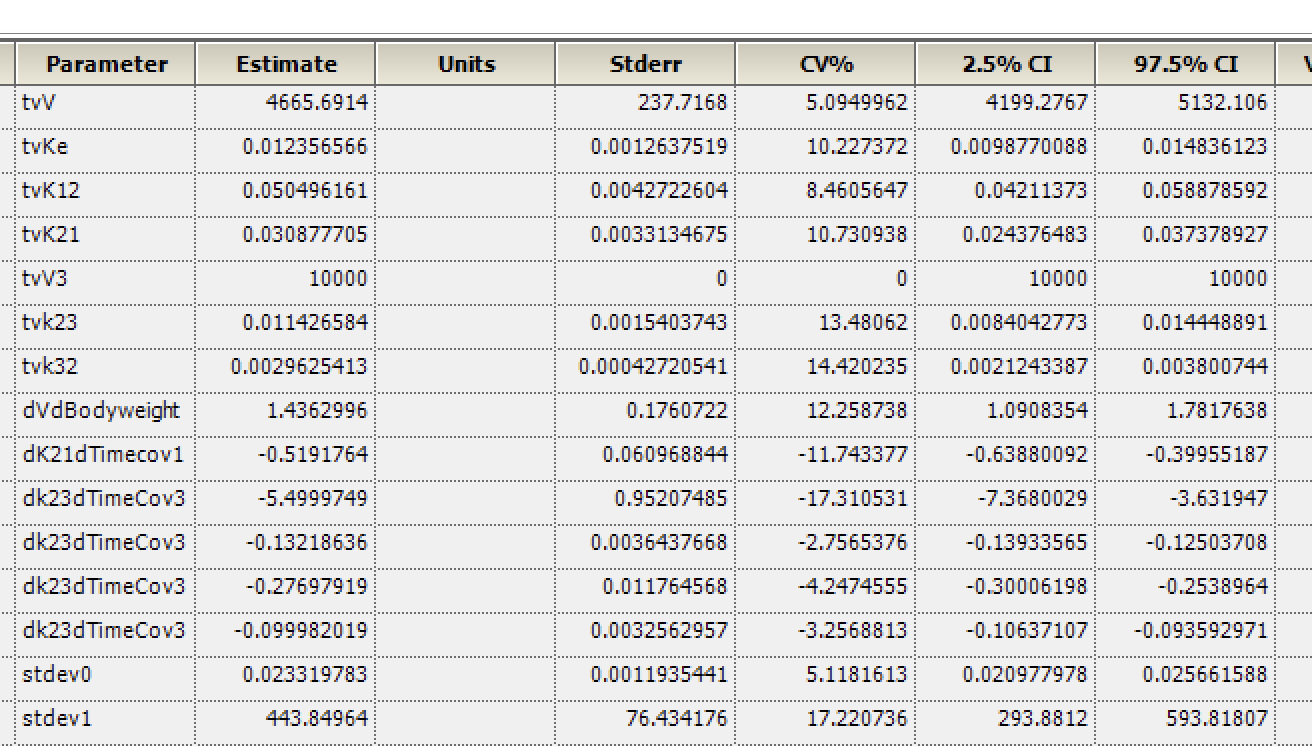


The TimeCov symbols are the same as for 1,000-2,000 mL (see above).

**10. 15-min infusions (awake) > 1 L infused**

29 infusions; age 32 (6), Sex 66% males, BW. 79 (14)

Hb 139 (13) inf vol 1941 (342). Inf time 15 (2) min, rate 125 (22) mL min^-1^

Data are the mean (SD).

516 data points. 41 urine outputs, total urine volume 923 (358) mL.

Akaike criterion 3-volume model -1532 and 2-volume model -1530

**
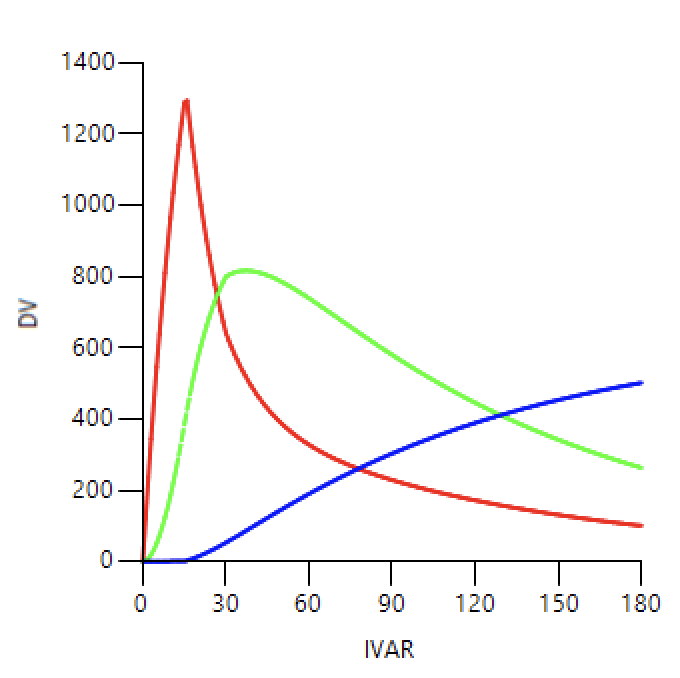
**

DV = volume expansion, IVAR = time (min), Red color = *V*_c_, Green color = *V*_t1_, Blue color = *V*_t2_


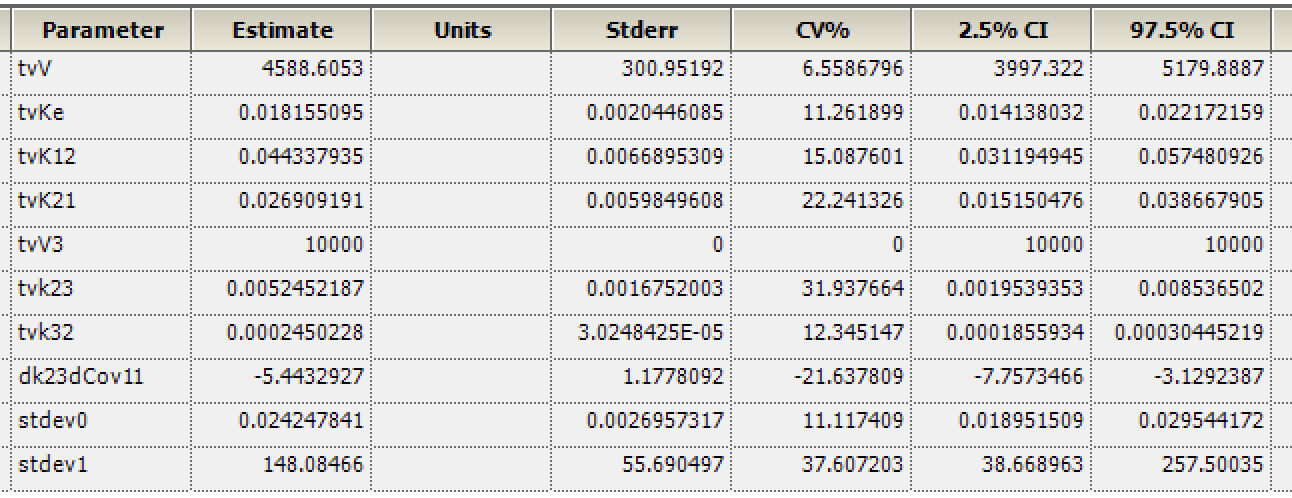


Cov 11: during infusion

**11. Infusion duration 45-80 min (awake)**

17 infusions; age 33 (7), Sex 47% males, BW 70 (12), Hb 131 (9)

inf vol 1321 (355). Inf time 51 (14) min, rate 26 (7) mL min^-1^ (data are the mean (SD)).

384 data points. 17 urine outputs, total urine volume 764 (424) mL.

3-volume model does not converge.

**
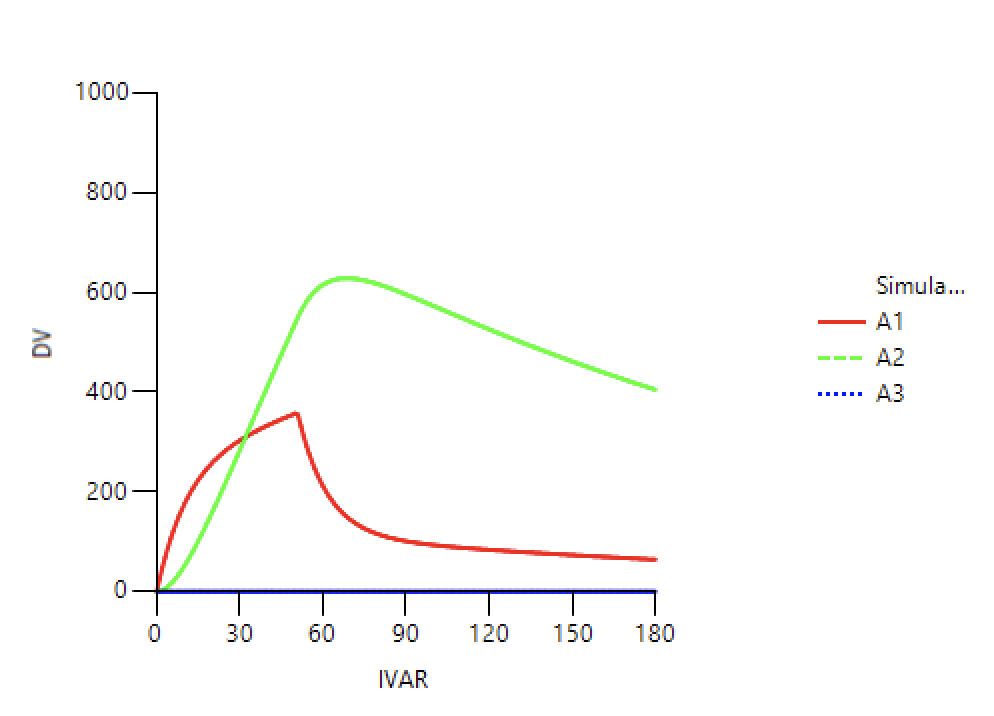
**

y-axis = volume expansion (mL), IVAR = time (min), Red color = *V*_c_, Green color = *V*_t1_


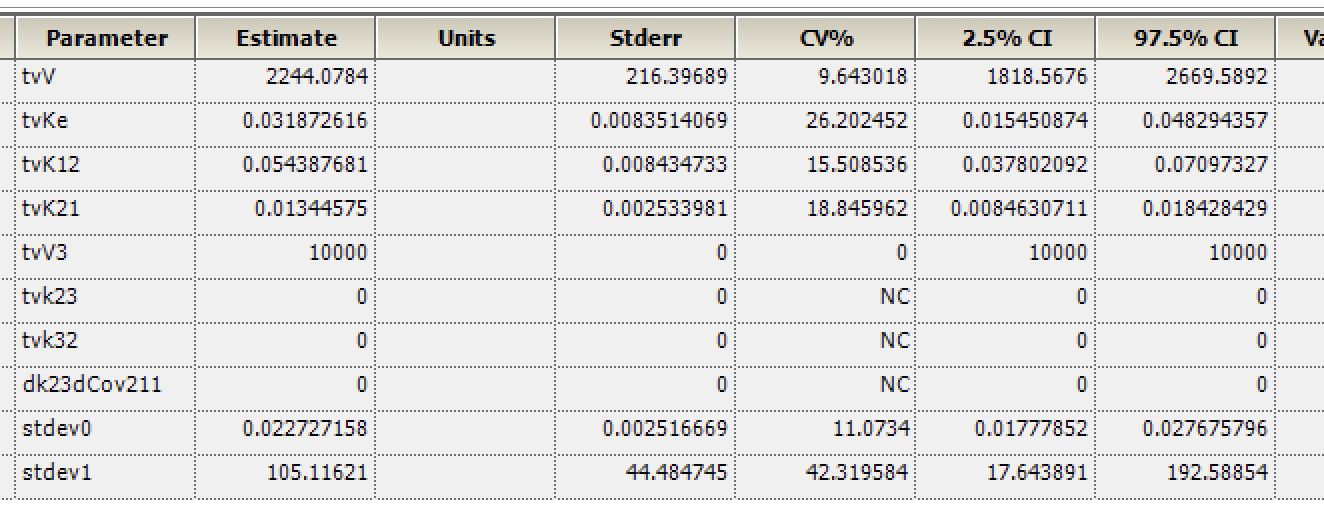


**
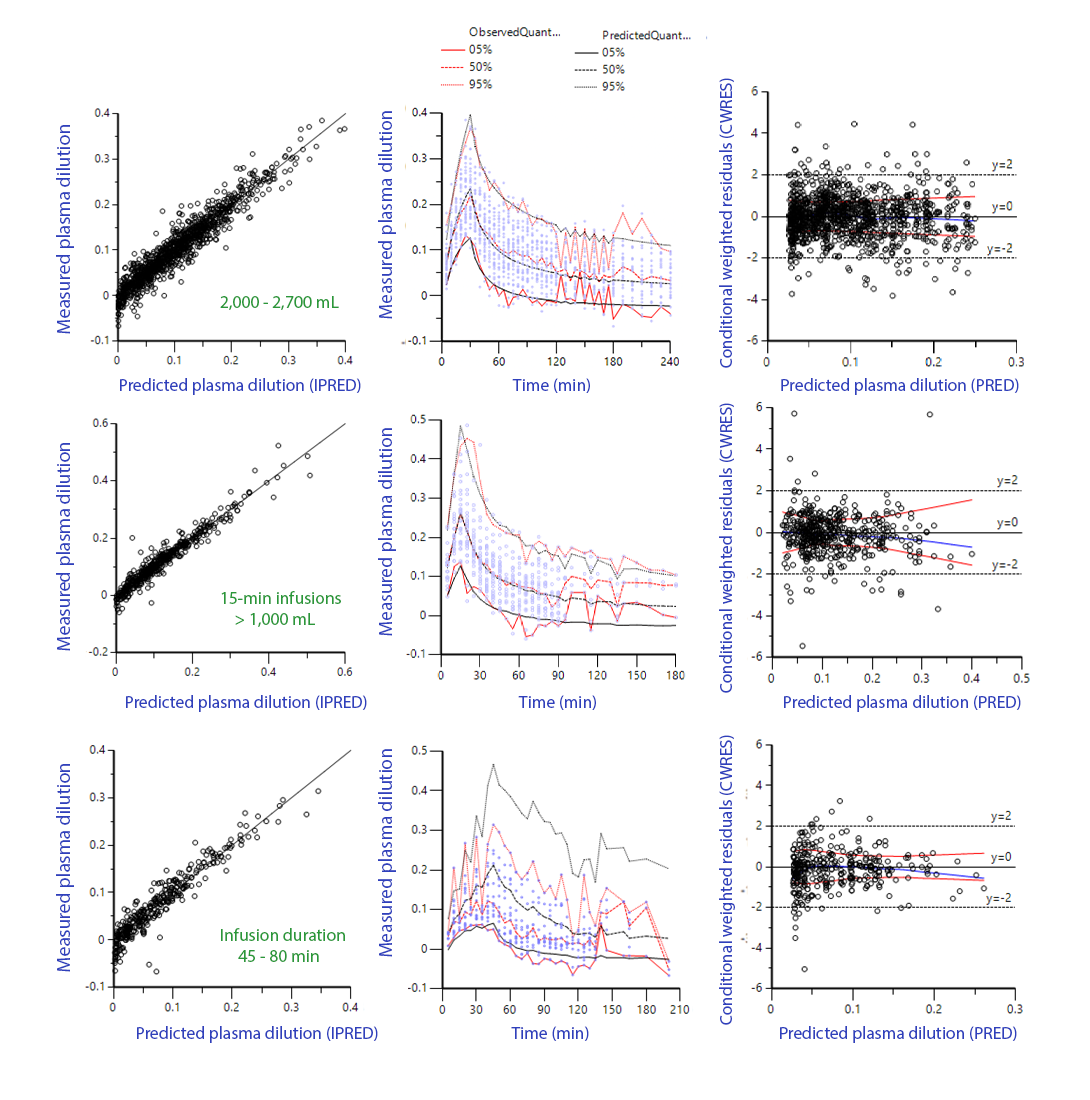
**

**12. GRAPHICAL PERFORMANCE MEASURES 2**

top: 2,000 mL, middle: 15-min infusions, and bottom: infusion duration 45-80 min

**Left column:** Measured plasma dilution *versus* the predicted dilution (with covariates).

**Middle column:** Predictive check. The measured plasma dilution and its 95% confidence interval is compared to the confidence interval for 1,000 simulations based in the optimal model parameters.

**Right column:** The conditional weighted residuals (CWRES) *versus* the predicted plasma dilution (without covariates).

**13. General anaesthesia**

54 infusions; age 50 (12), Sex 8% males, BW. 71 (12)

Hb 120 (13) inf vol 1779 (316). Inf time 30 min, rate 59 (11) mL min^-1^

930 data points. 210 urine outputs (only the final sum was used).

Total urine output median 73 mL (interquartile range 50-181).

Akaike criterion 3-volume model -2438 and 2-volume model -2336


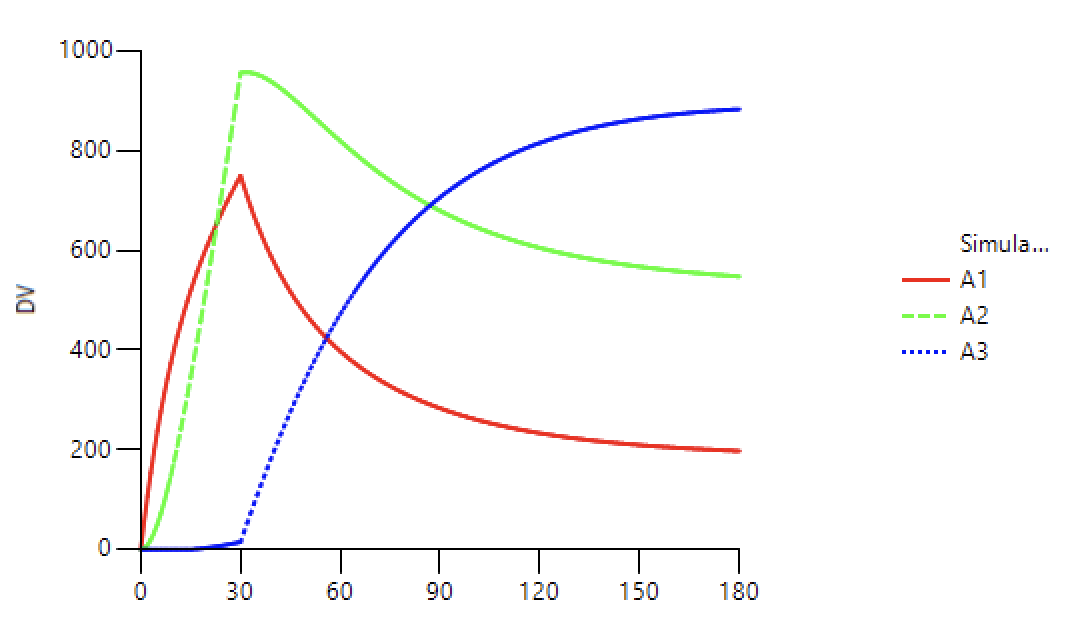


DV = volume expansion (mL), x-axis = time (min), Red color = *V*_c_, Green color = *V*_t1_, Blue color = *V*_t2_


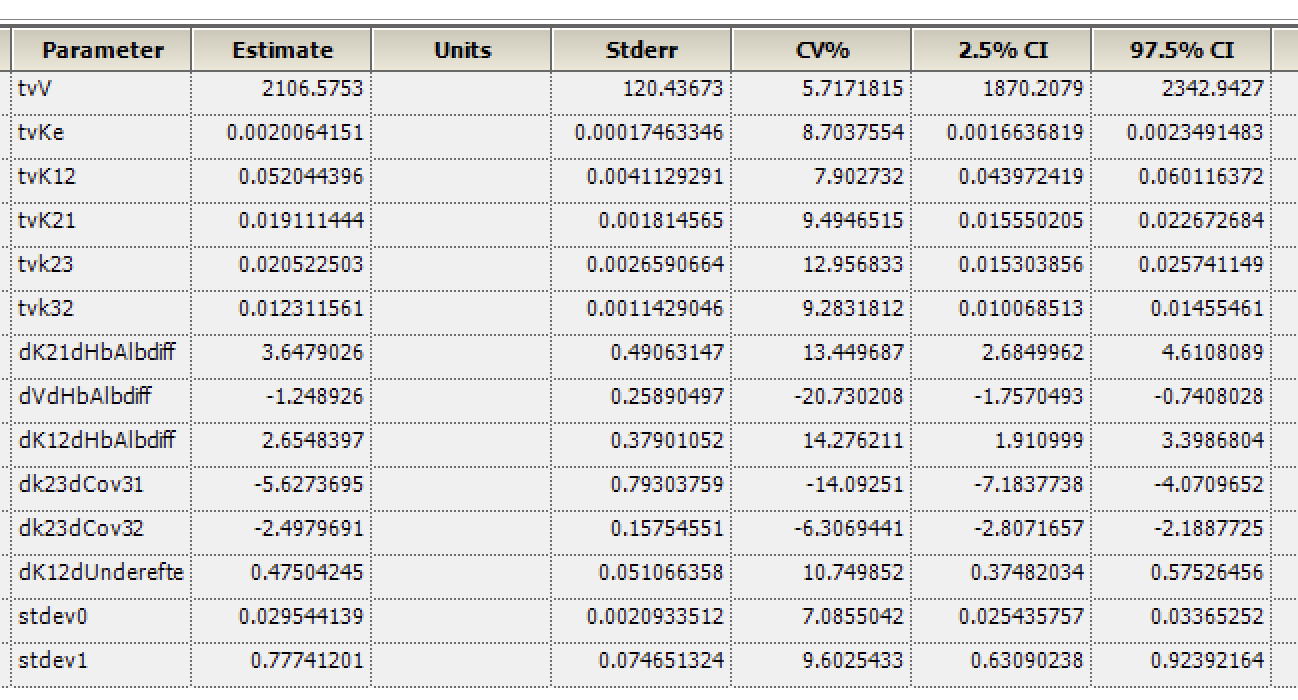


The Hb-Albumin dilution difference was used as time-varying covariate to correct for interstitial washdown. Cov 31: 0-15 min of the infusion., Cov 32: 15-30 min of the infusion (to the end)

**14. Haemorrhage**

20 infusions given immediately after 450 mL (N=10) and 900 mL (N=10) of blood had been withdrawn; age 28 (12), Sex 100% males, BW 75 (7)

inf vol 1841 (136). Inf time 30 min, rate 61 (5) mL min^-1^

463 data points. 20 urine measured outputs, total urine output 737 (428) mL.

Data are the mean (SD).

Akaike criterion 3-volume model -1599 and 2-volume model -1592

**
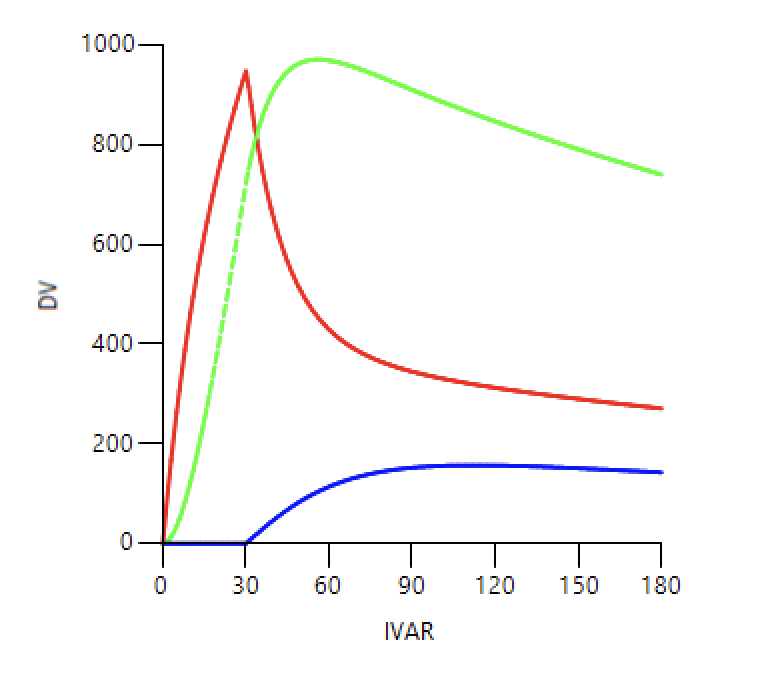
**

DV = volume expansion (mL), IVAR= time (min), Red color = *V*_c_, Green color = *V*_t1_, Blue color = *V*_t2_


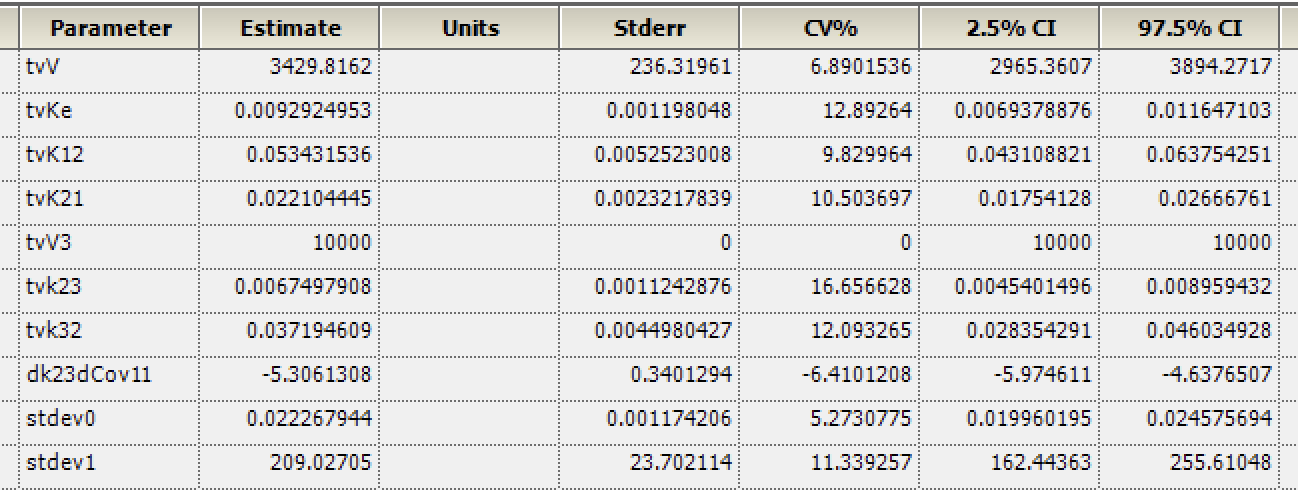


The data for this analysis was taken from the following two publications:

Ewaldsson CA, Hahn RG. Kinetics and extravascular retention of acetated Ringer’s solution during isoflurane and propofol anesthesia for thyroid surgery. Anesthesiology. 2005:103:460-469.

Hahn RG, Nemme J. Volume kinetic analysis of fluid retention after induction of general anaesthesia. BMC Anesthesiology 2020;20:95.

**15. Inflammation**

40 infusions; age 44, Sex 40% males, BW 58 (11) kg.

inf vol 869 (168). Inf time 35 min, rate 25 (5) mL min^-1^

753 data points. 40 urine outputs, total urine output 118 (105) mL.

Data are the mean (SD).

Akaike criterion 3-volume model -1122 and 2-volume model -1117

**
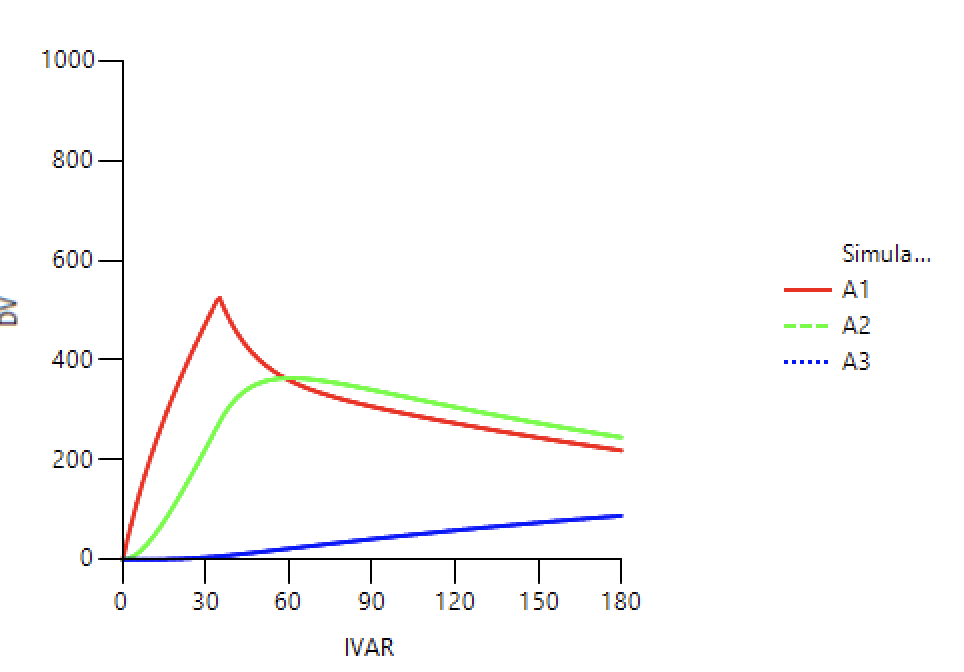
**

y-axis = volume expansion (mL), IVAR= time (min), Red color = *V*_c_, Green color = *V*_t1_, Blue color = *V*_t2_


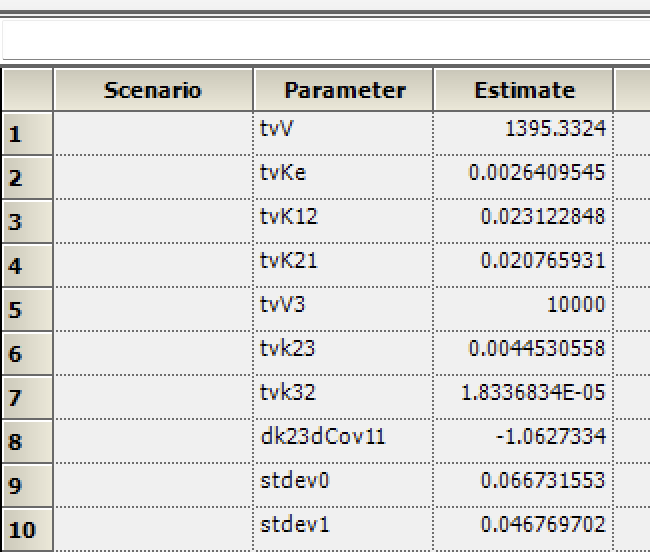


The data for this analysis was taken from the following publication:

Li Y, Yi S, Zhu Y, Hahn RG. Volume kinetics of Ringer’s lactate solution in acute inflammatory disease.

Br J Anaesth 2018; 121: 574-580.

**
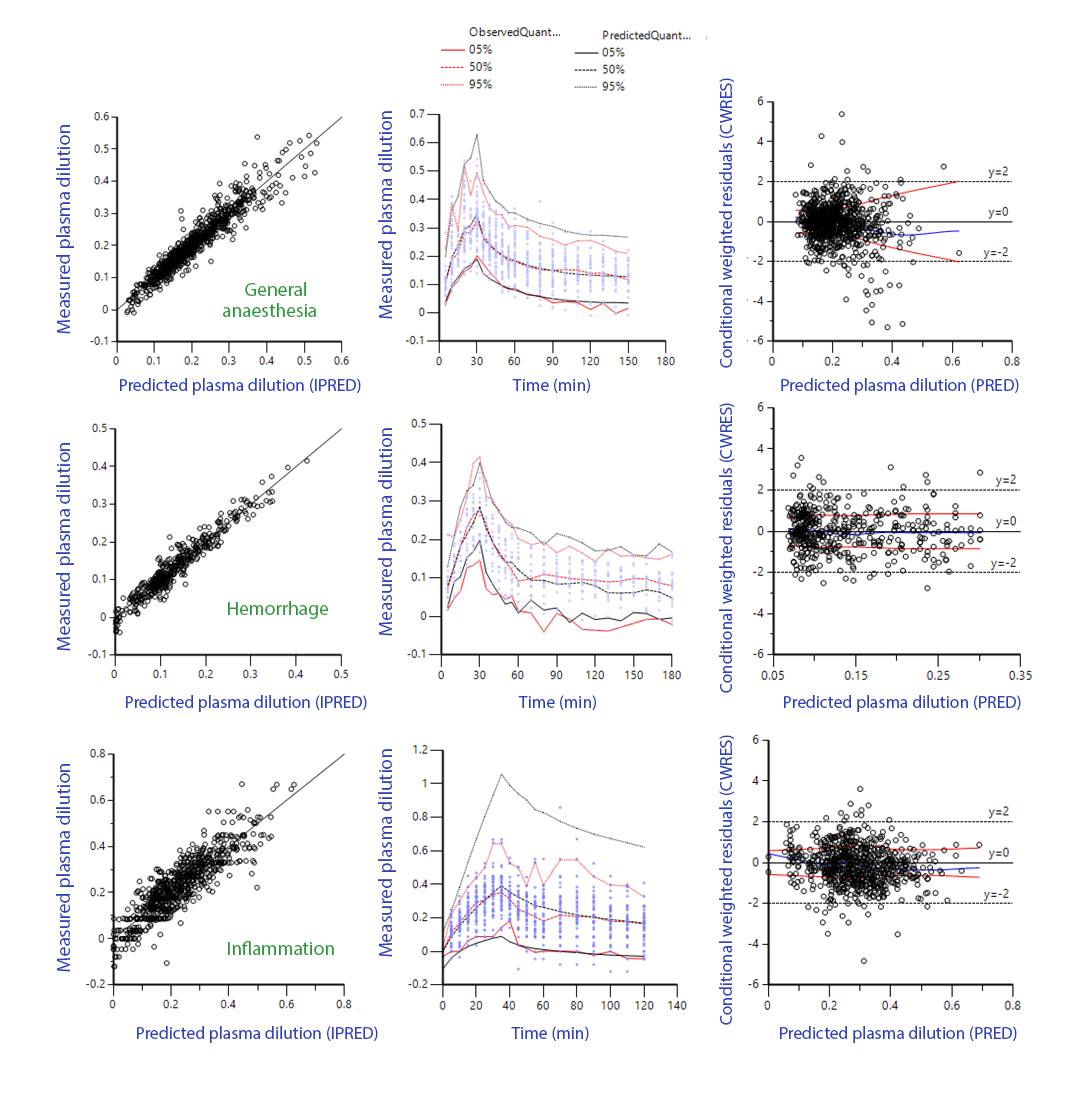
**

**16. GRAPHICAL PERFORMANCE MEASURES 3**

top: General anethesia, middle: Hemorrhage, and bottom: Inflammation

**Left column:** Measured plasma dilution *versus* the predicted dilution (with covariates).

**Middle column:** Predictive check. The measured plasma dilution and its 95% confidence interval is compared to the confidence interval for 1,000 simulations based in the optimal model parameters.

**Right column:** The conditional weighted residuals (CWRES) *versus* the predicted plasma dilution (without covariates).

**
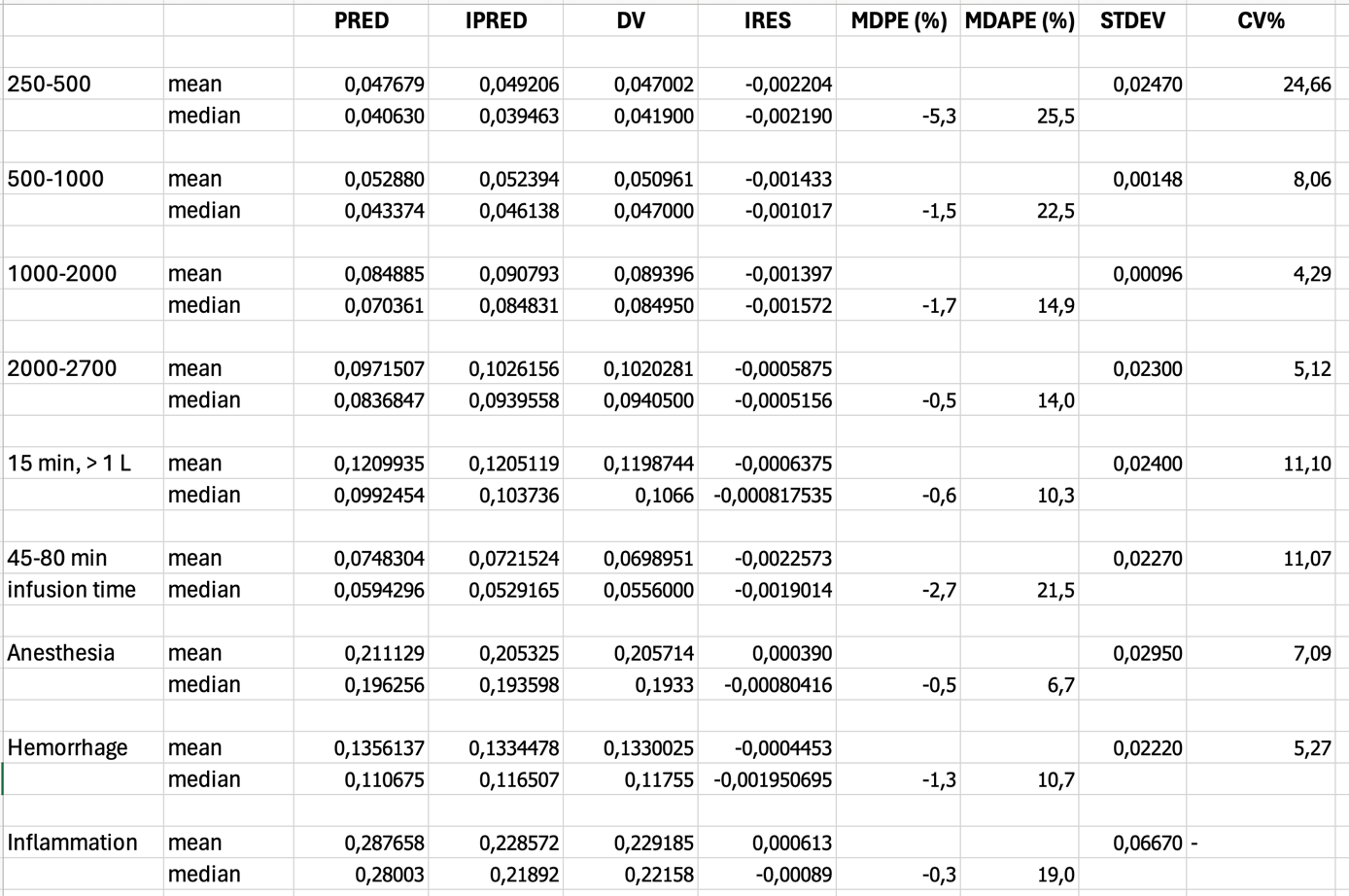
**

**17. Table S2. TABULATED PERFORMANCE MEASURES**

Shown are performance measures for all 9 subgroups.

PRED = plasma dilution predicted by the kinetic model (no covariates)

IPRED = plasma dilution predicted by the kinetic model (with covariates)

DV = measured plasma dilution

IRES (residual) = difference between DV and IPRED

MDPE = median prediction error, the median value of IRES / IPRED (the bias).

MDAPE = absolute value of MDPE (the precision)

STDAV = standard error of the estimated IRES as given by the Pheoenix program

CV%= between-subject variability in STDAV as given by the Pheoenix program

The use of MDPE and MDAPE is explained in:

Varvel JR, Donoho DL, Shafer SL. Measuring the predictive performance of computer-controlled infusion pumps. J Pharmacokinet Biopharm. 1992; 20:6 3-94.

**18. Phoenix program file**

Phoenix Program file used for simulation of the plot for the infusion of 1,000-2,000 mL over 30 min in awake volunteers. The basic differential equations for the kinetic model are highlighted by red color. Ke = *k*_10_. The “sleep” lines instruct the program to change parameter value after the time shown in parenthesis.

test(){

deriv(A1 = - (A1 * Ke)- (A1 * K12- A2 * K21))

urinecpt(A0 = (A1 * Ke))

deriv(A2 = (A1 * K12- A2 * K21)- (A2 * k23- A3 * k32))

deriv(A3 = (A2 * k23- A3 * k32))

double(tvK21)

sequence{

tvK21=0.0101

sleep(30)

tvK21=0.030

}

double(tvk23)

sequence{

tvk23=0.00051

sleep(20)

tvk23=0.001756

sleep(10)

tvk23=0.0153

sleep(20)

tvk23=0.017

}

C = A1 / V

dosepoint(A1, idosevar = A1Dose, infdosevar = A1InfDose, infratevar = A1InfRate)

error(CEps = 0.0268931440925238)

observe(CObs = C + CEps)

error(A0Eps = 96.5731390598463)

observe(A0Obs = A0 + A0Eps)

C3 = A3 / V3

stparm(V = tvV * exp(nV))

stparm(Ke = tvKe * exp(nKe))

stparm(K12 = tvK12 * exp(nK12))

stparm(K21 = tvK21 * exp(nK21))

stparm(V3 = tvV3 * exp(nV3))

stparm(k23 = tvk23 * exp(nk23))

stparm(k32 = tvk32 * exp(nk32))

fixef(tvV = c(, 4155, ))

fixef(tvKe = c(, 0.01676, ))

fixef(tvK12 = c(, 0.0463, ))

fixef(tvV3 = c(, 10000, ))

fixef(tvk32 = c(, 0.007256, ))

ranef(diag(nV, nKe, nK21, nK12, nV3, nk23, nk32) = c(0.30355015, 1.4954665, 0.173917, 0.2311694,
